# Supplementary material for: Nonhuman primate antigenic cartography of SARS-CoV-2
Source: Cell Rep. 2025 Jan 3;44(1):115140. doi: 10.1016/j.celrep.2024.115140 (PMC11781863; doi:10.1016/j.celrep.2024.115140)
Supplement: Document S1. Figures S1–S10 and Tables S1–S3, S5, and S6 [file mmc1.pdf]

Cell Reports, Volume *44*

Supplemental information

Nonhuman primate antigenic cartography of SARS-CoV-2

Annika Rössler, Antonia Netzl, Ninaad Lasrado, Jayeshbhai Chaudhari, Barbara Mühlemann, Samuel H. Wilks, Janine Kimpel, Derek J. Smith, and Dan H. Barouch

Supplementary Figures

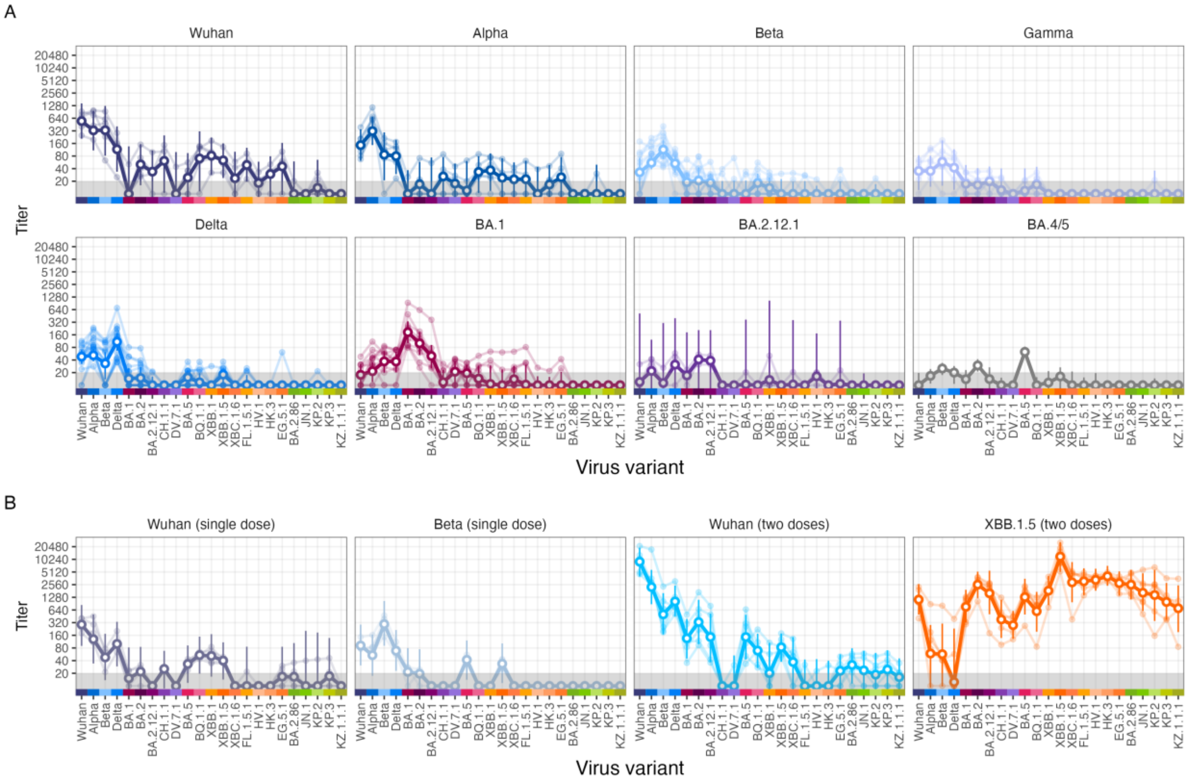

Figure S1. Titers with reactivity adjustment, related to Figure 2. A. Infected study cohorts by infecting virus variant and **B.** vaccine cohorts by vaccine regimen. Individual samples are shown as transparent lines with the geometric mean titer (GMT) given as solid line and the bars indicating 95% CI. The colors correspond to the colors in the map, the variant colors are given on the x axis. The numbers on top of each panel give the GMT against the respective variant in the top row and fold change from homologous in the bottom row. The GMT and fold changes were calculated using the titertools R package [1] (v.0.0.0.9001). Reactivity reduction of the Alpha variant was performed as described in the methods section; the raw Alpha titers were multiplied by a factor of 0.58.

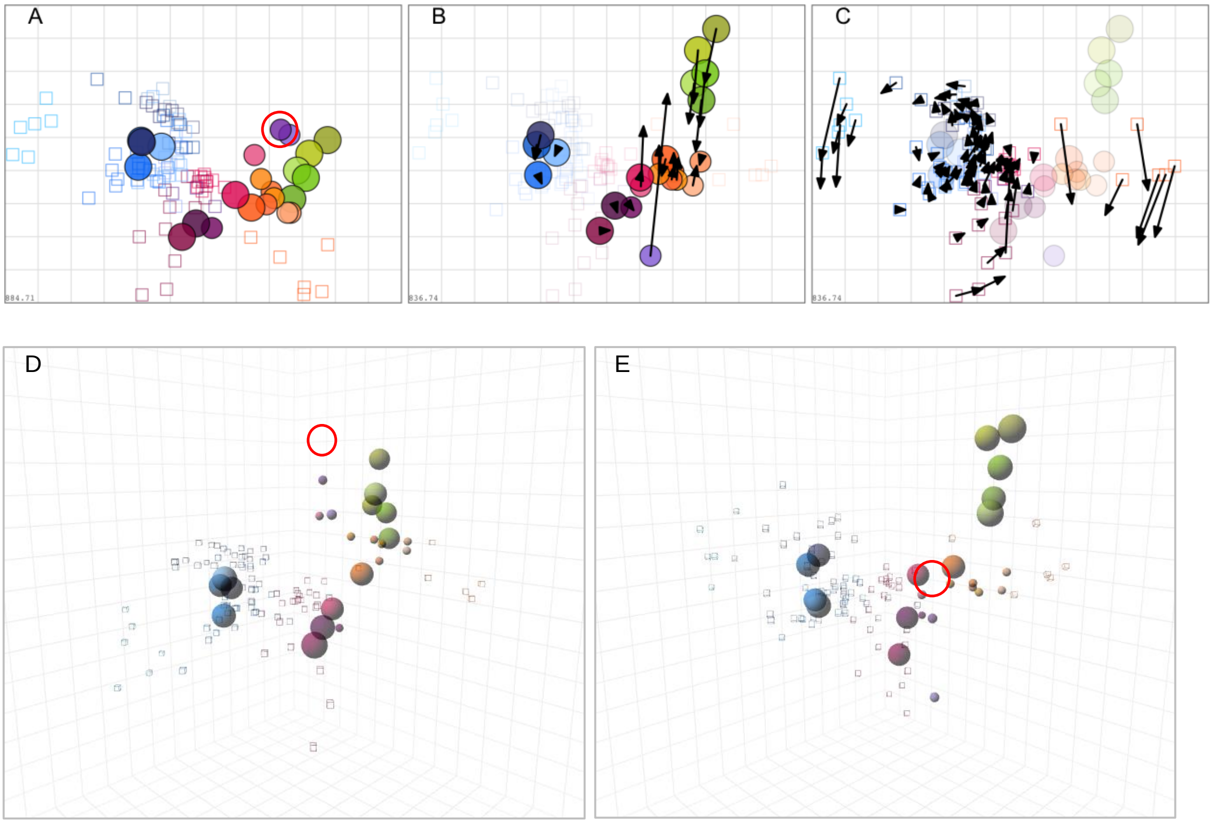

Figure S2. Bistable map including CH.1.1, related to Figure 3. A. A map including the highlighted CH.1.1 was constructed as described in the methods section, resulting in a different optimum. **B.** Arrows point from the variant's position in the map in fig.3A to the variant's position in the map in A. **C.** Arrows point from serum's position in the map in fig.3A to the variant's position in the map in A. **D, E.** The map in A was optimized in three dimensions and is shown in two different orientations. **D.** Shows a 3D orientation that would result in a map conformation as in **A** if projected onto 2D., **E.** shows a 3D orientation that would result in a map conformation as in **B** if projected onto 2D.

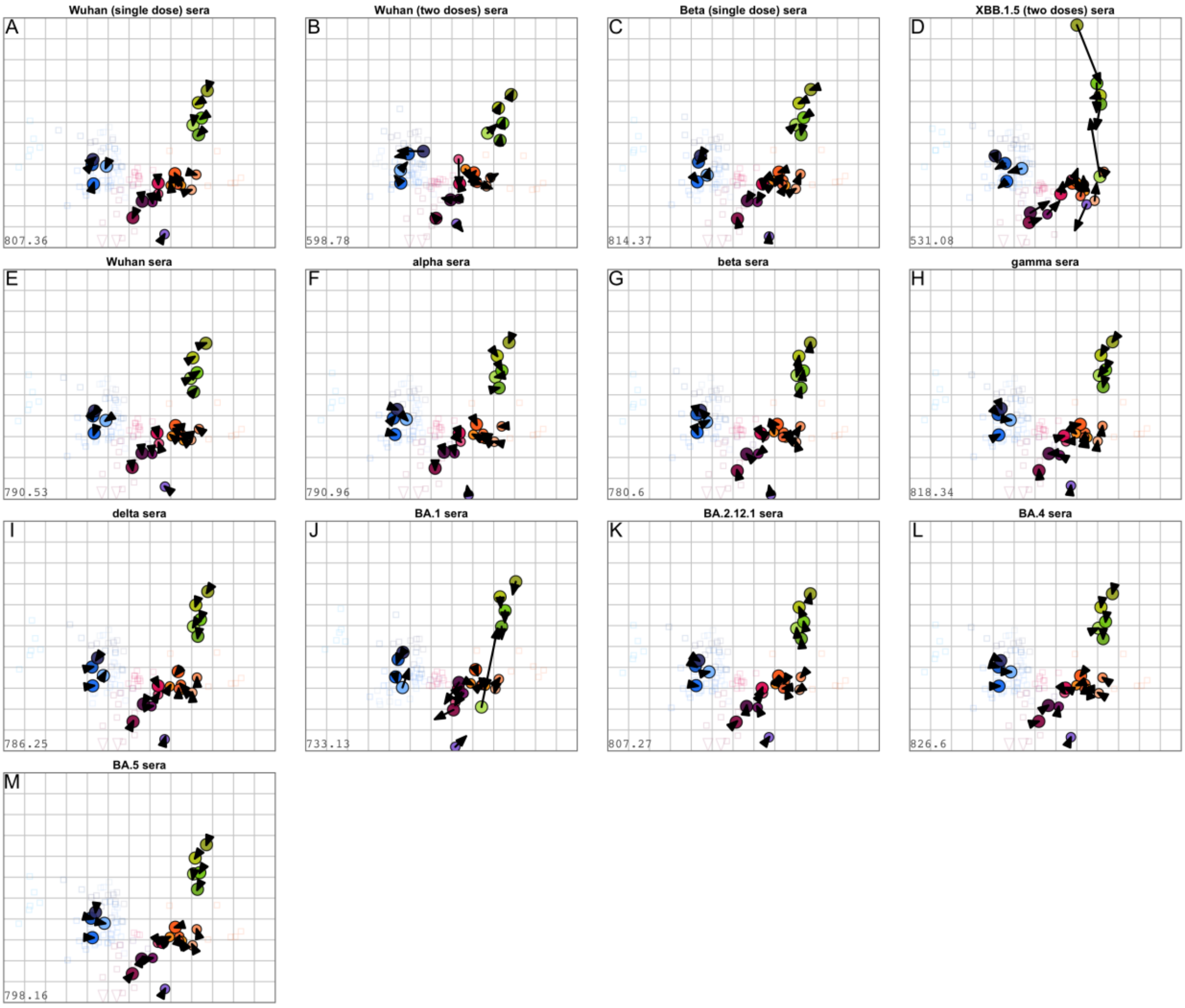

Figure S3. Assessing map robustness to the exclusion of sera, related to Figure 3. Each serum group was removed and the map reoptimized, with optimization settings as described in the Methods section. Arrows point to the position of each variant in the map shown in **figure 3**, for color correspondence refer to this map. A small arrow length indicates similar variant positions and map robustness to the exclusion of the particular serum group. Maps without sera vaccinated with **A.** Wuhan (single dose), **B.** Wuhan (two doses), **C.** Beta (single dose), **D.** XBB.1.5 (two doses), and infected with **E.** Wuhan, **F.** alpha, **G.** beta, **H.** gamma, **I.** delta, **J.** BA.1, **K.** BA.2.12.1, **L.** BA.4, or **M.** BA.5.

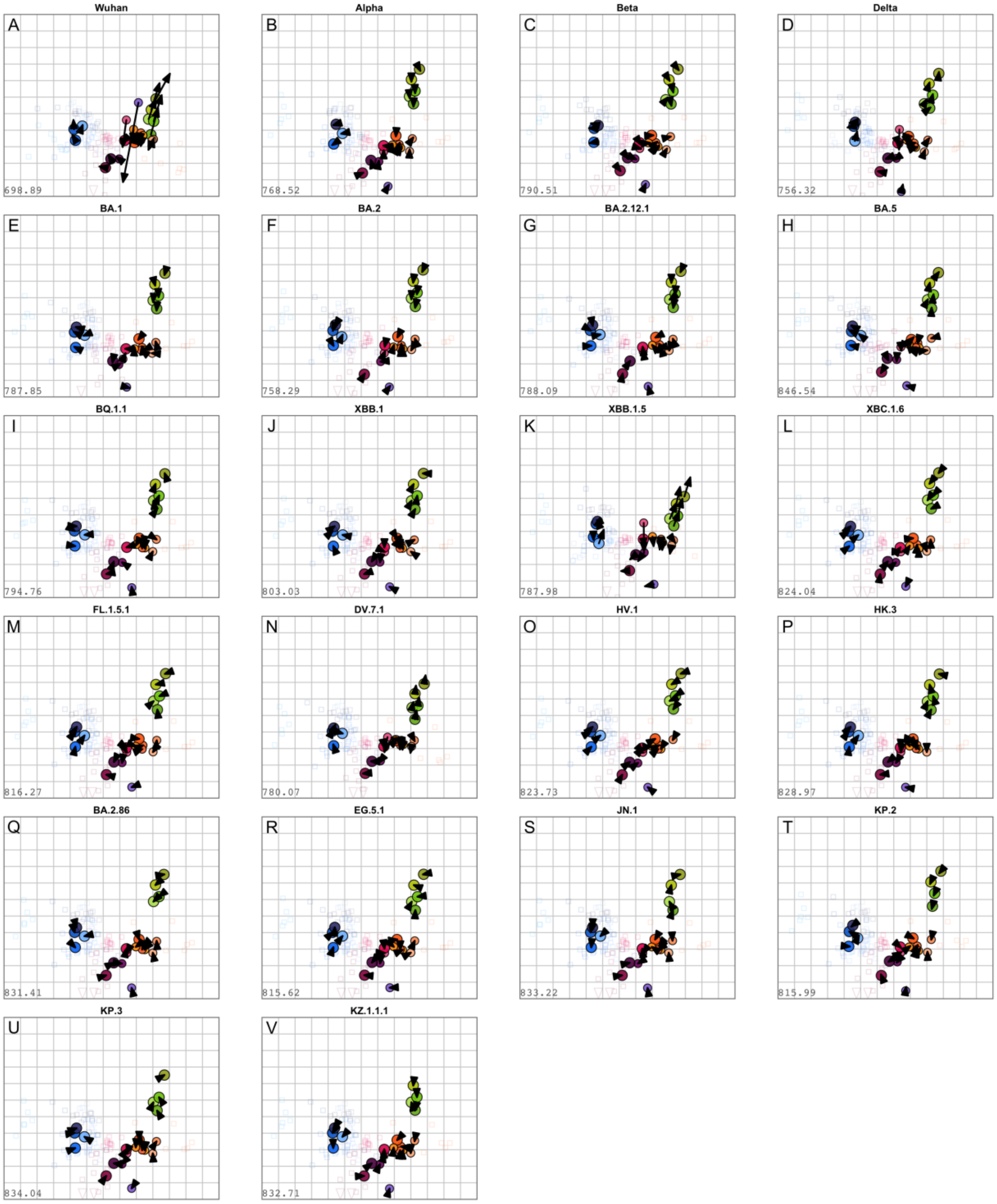

Figure S4. Assessing map robustness to the exclusion of antigen variants, related to Figure 3. Each antigen variant was removed and the map reoptimized, with optimization settings as described in the Methods section. Arrows point to the position of each variant in the map shown in **figure 3**, for color correspondence refer to this map. A small arrow length indicates similar variant positions and map robustness to the exclusion of the particular variant.

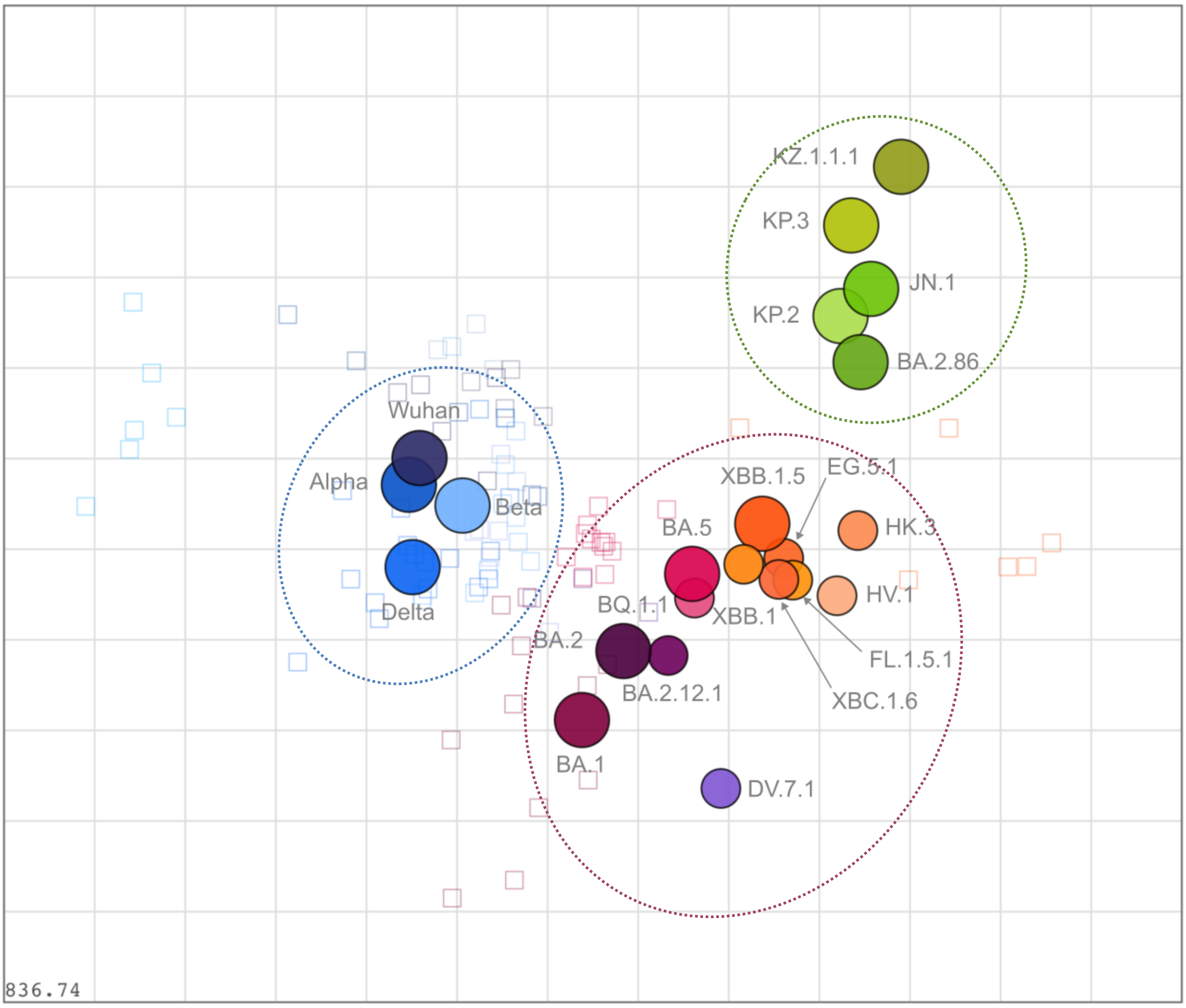

Figure S5. Zoomed-out version of the antigenic map, related to Figure 3. Virus variants are shown as colored circles, smaller circles denote sub-lineages, sera as open squares with the color corresponding to the infecting variant, vaccine sera are shown in grey tones. Dashed lines indicate three virus clusters; we grouped them according to their map position and clusters are highlighted by blue, purple/orange and green color schemes. The x- and y-axis represent antigenic distances with one grid square corresponding to one two-fold serum dilution of the neutralization titer. The map orientation within x- and y-axis is free as only relative distances can be inferred. Only single variant exposure sera and double vaccination sera have been used for construction of the map, the number of which is given in the legend of **figure 3**.

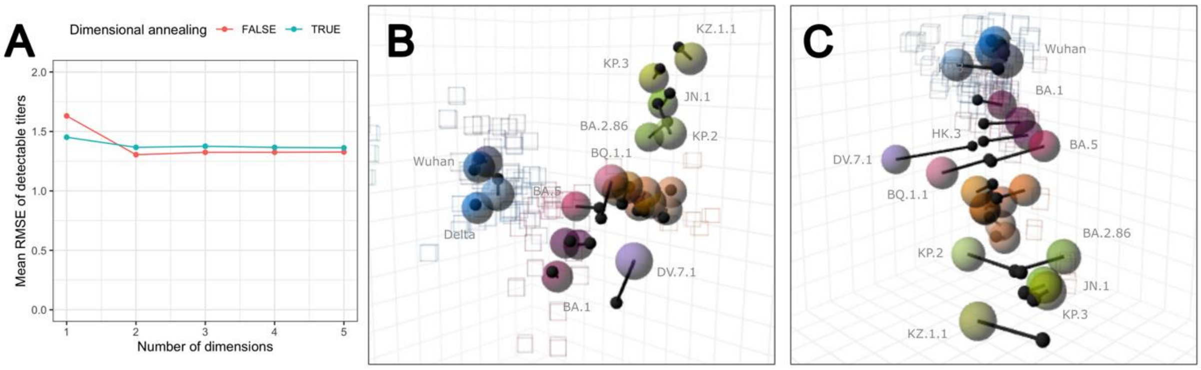

Figure S6. Map dimensionality, related to Figure 3. A Dimensionality test: Root Mean Squared Error (RMSE) between map and measured titers for detectable titers in 1 to 5 dimensions. Per dimension, 100 map replicates were constructed from 90% of measured titers with 1000 optimizations per replicate, once with the option "dim_annealing = TRUE" (blue) and once with "dim_annealing = FALSE" (red). The titers of the remaining 10% were predicted in each run and the RMSE calculated by comparing the predicted to the measured titers on the $\log_2$ scale. Both options were tested as the dimensional annealing with missing data did not find the global optimum, resulting in extremely similar RMSE across all dimensions [2]. **B, C** Front and side view of the map optimized in 3 dimensions with arrows pointing to the variants' position in the 2D map ("dim_annealing = TRUE").

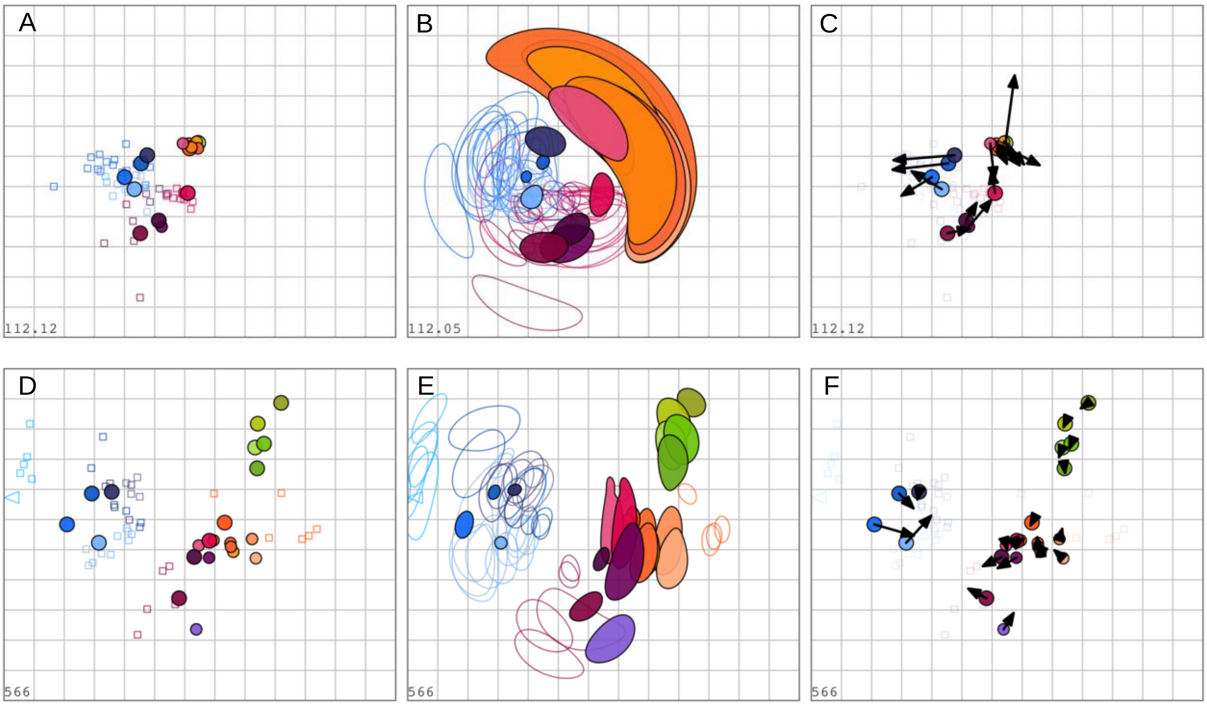

Figure S7. Antigenic maps from early and later sampled sera, related to Figure 3. Maps were constructed from sera sampled **A.** up to D10 post challenge and **D.** later than D10 post challenge. **B, E.** Constant force loci show the area which an item can occupy in the respective map without increasing map stress by more than one unit. **C, F.** Arrows point to the variants' positions in the main map. Descriptions apply to the map in the respective row.

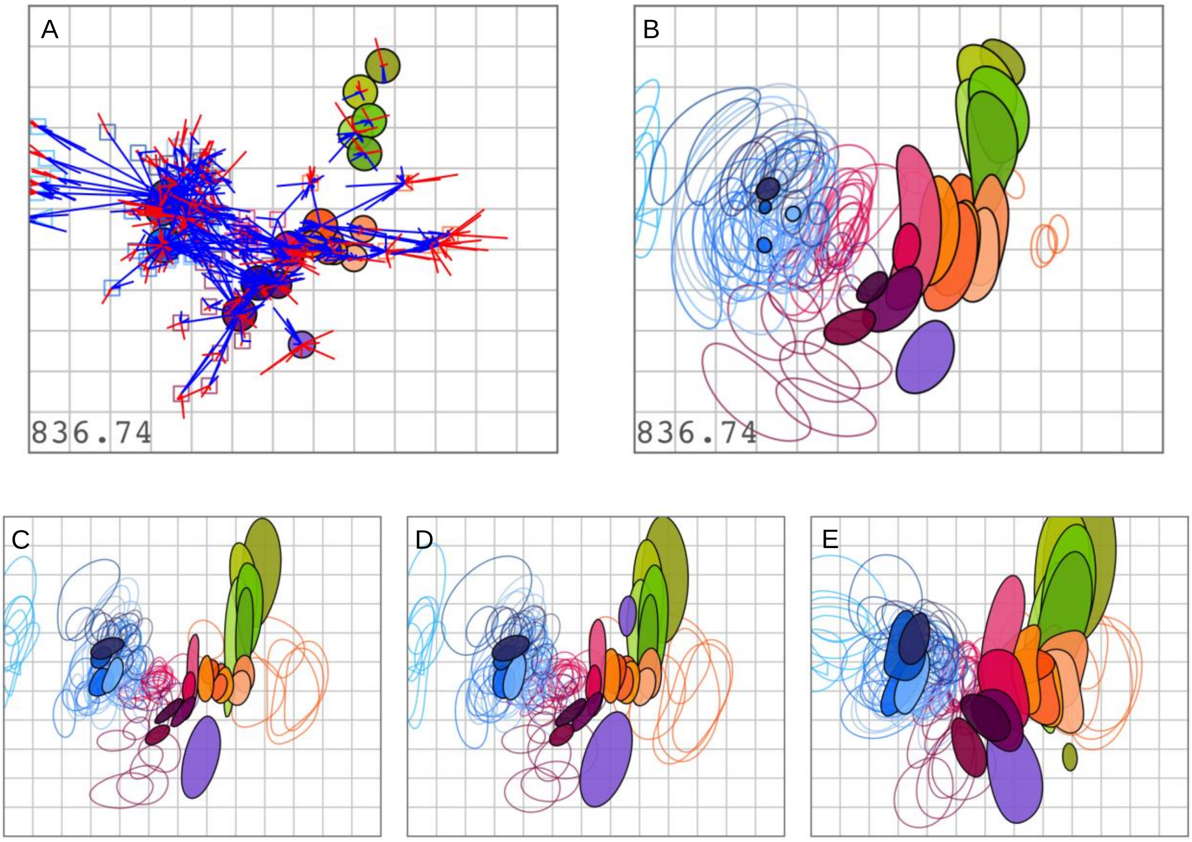

Figure S8. Map error and positional resolution, related to Figure 3. A. Error lines for each serum and antigen are shown in blue in case of larger map distance than target distance and red in case of smaller map distance than target distance. The length of each error bar indicates the magnitude of mismatch. Blue error lines point towards the variant-serum pair that has a smaller target distance; red error lines point away from the variant-serum pair. **B.** Constant force loci (Triangulation blobs) show the area for each serum and variant in which the item can move without increasing map stress by more than one unit. Filled shapes show variant Triangulation blobs, open shapes sera. **C-D.** 500 bootstrap repeats were performed with 1000 optimizations per repeat and options as listed in the Methods section. In each repeat, **C.** different weights are assigned to sera and antigen reactivity of the titer table. The weights are drawn randomly from a Dirichlet distribution. **D.** Sera and antigens are randomly resampled with replacement, and **E.** normally distributed noise with sd = 0.7 is added to antigens and individual titer measurements. The colored regions mark 68% (one standard deviation) of the positional variation for each variant (filled shapes) and sera (open shapes). The colors correspond to the colors used in **figure 3**.

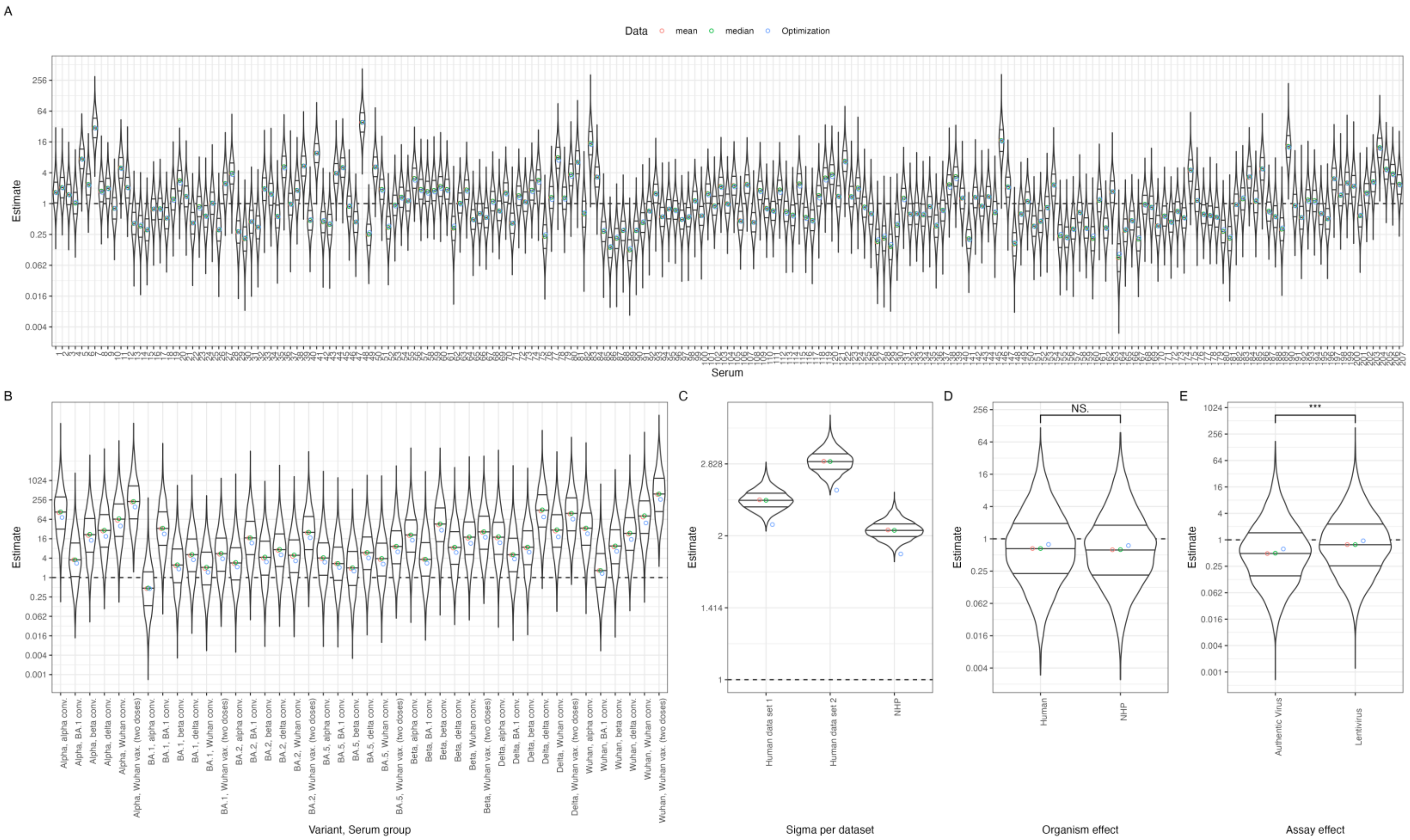

Figure S9. Bayesian modelling estimates, related to Figure 4. Estimated **A.** serum reactivity effect, **B.** underlying variant titer/10 per serum group, **C.** normally distributed error (sigma) per dataset (Human data set 1 [3], Human data set 2 [4] and NHP), **D.** organism and **E.** assay effects from 4000 independent draws. The mean and median of the draws are shown in red and green, respectively, and the mean from optimizing the model is shown in blue. **Table S4** links serum number in **A.** and serum name. Estimated **D.** organism and **E.** assay effects from independent were compared using a T-test, normality was checked with a Shapiro-Wilk test (Human p=0.224, NHP p=0.352, Authentic virus p=0.085, Lentivirus p=0.304). No significant difference was detected for organism effects (statistic = 1.52, df = 7998, p = 0.128). The difference between assay effects was highly significant (statistic = -13, df = 7988, p = 2.78e-38).

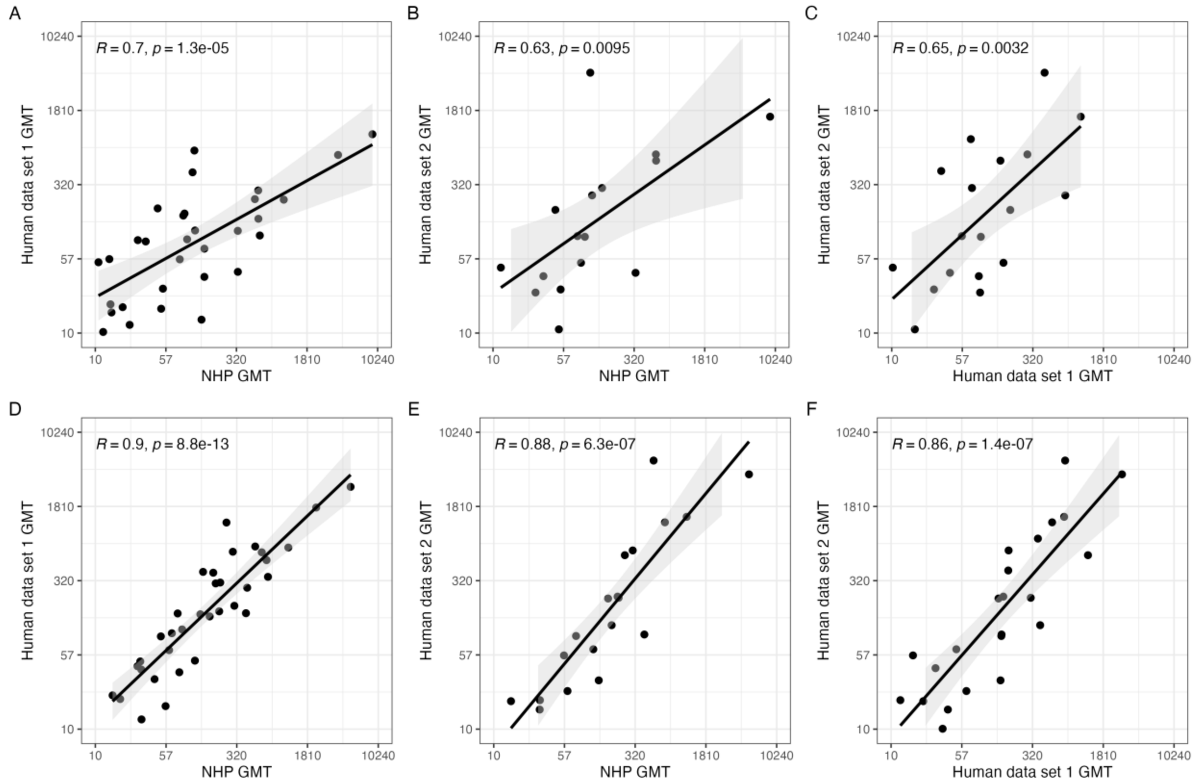

Figure S10. GMT correlation of data sets, related to Figure 4. A-C Correlation of GMTs calculated from imputed titers across datasets before adjusting for assay and organism effects and **D-E** after adjusting for assay and organism effects. The Pearson correlation coefficient is shown for NHP vs. Human data set 1 [3] (**A, D**), NHP vs. Human data set 2 [4] (**B, E**) and Human data set 1 vs. Human data set 2 (**C, F**).

Supplementary Tables

Table S1. SARS-CoV-2 variants analyzed in the pseudovirus neutralization assay, related to Figure 1

**Category**	**Virus variant (Pango lineage)**	**GISAID ID†**
Pre-Omicron variants	Wuhan (WA1/2020)	EPI_ISL_402124
	B.1.1.7	EPI_ISL_601443
	B.1.351	EPI_ISL_712096
	B.1.617.2	EPI_ISL_2020950
Early Omicron variants	BA.1	EPI_ISL_7358094.2
	BA.2	EPI_ISL_6795834.2
	BA.2.12.1	EPI_ISL_12003853.1
	CH.1.1	EPI_ISL_17202004
	DV.7.1	EPI_ISL_18052118
	BA.5	EPI_ISL_12268495.2
	BQ.1.1	EPI_ISL_14752457
XBC.1.6 and XBB.1-descended variants	XBB.1	EPI_ISL_15232105
	XBB.1.5	EPI_ISL_16418320
	XBC.1.6	EPI_ISL_17851490
	FL.1.5.1	EPI_ISL_18126515
	HV.1	EPI_ISL_18592608
	HK.3	EPI_ISL_18631954
	EG.5.1	EPI_ISL_17976635
Recent saltation Omicron variants	BA.2.86	EPI_ISL_18121060
	JN.1	EPI_ISL_18680594
	KP.2	EPI_ISL_19189039
	KP.3	EPI_ISL_19203968
	KZ.1.1.1	EPI_ISL_19028189

†The spike sequences for the pseudovirus generation were retrieved from the GISAID Initiative [5].

Table S2. Characteristics of infected NHP study cohorts, related to Figures 1 and 2

Study cohort	Virus variant of challenge (Pango lineage)	SARS-CoV-2 isolate	GISAID ID	Number of animals (n)	Blood collection*
**Wuhan**	Wuhan	hCoV-19/USA-WA1/2020	EPI_ISL_404895.2	5	20#
**Alpha**	B.1.1.7	hCov-19/USA/CA_CDC_5574/2020	EPI_ISL_751801	6	21#
**Beta**	B.1.351	hCov-19/South Africa/KRISP-K005325/2020	EPI_ISL_678615	12	10 (n=6)<br>21 (n=6#)
**Gamma**	P.1	hCoV-19/Japan/IC-0564/2021	EPI_ISL_792683	6	10
**Delta**	B.1.617.2	hCoV-19/USA/MD-HP05647/2021	EPI_ISL_2331496	16	10
**BA.1**	B.1.1.529	hCoV-19/USA/MD-HP20874/2021	EPI_ISL_7160424	11	10 (n=6)<br>28 (n=5#)
**BA.2.12.1**	BA.2.12.1	hCoV-19/USA/NY-MSHSPSP-PV56475/2022	EPI_ISL_11685455	3	10
**BA.4**	BA.4	hCoV-19/USA/MDHP30386/2022	EPI_ISL_12416220	3	10
**BA.5**	BA.5	hCov-19/USA/COR-22-063113/2022	EPI_ISL_13512579	9	10

*Interval between virus challenge and blood collection is given in days.
#Additional samples from an earlier timepoint (day 10 post infection) have been analyzed (**figure S4**).

Table S3. Characteristics of vaccinated NHP study cohorts, related to Figures 1 and 2

Study cohort	Number of animals (n)	COVID-19 vaccine	Vaccine dose	Vaccine doses† (n)	Blood collection*
**Wuhan**	4	Ad.26.COV.S; Janssen COVID-19 viral-vector vaccine	$5x10^{10}$ Virus particles	1	26
**Beta**	4	Ad.26.COV.S.351; Janssen COVID-19 viral-vector vaccine	$5x10^{10}$ Virus particles	1	26
**Wuhan**	6	BNT162b2; Pfizer COVID-19 mRNA vaccine	30 ug mRNA	2	14
**XBB.1.5**	6	BNT162b2; Pfizer COVID-19 mRNA vaccine	30 ug mRNA	2	14

†Number of subsequent homologous vaccine doses.
*Interval between last vaccination and blood collection is given in days.

Table S5. Number of titrations per serum group, variant and data set used in the Bayesian comparison, related to Figure 4. Titrations from maps by Roessler *et al.* [3] (Human data set 1) and Wilks *et al.* [4] (Human data set 2) that were common in serum group and variant with the NHP data set were extracted. The D614G variant in the human data set was taken as Wuhan equivalent.

Serum group	Virus variant	Human data set 1	Human data set 2	NHP
BA.1 infected	Alpha	14	1	11
BA.1 infected	BA.1	14	4	11
BA.1 infected	BA.2	14	4	11
BA.1 infected	BA.5	14	4	11
BA.1 infected	Beta	14	4	11
BA.1 infected	Delta	14	1	11
BA.1 infected	Wuhan	14	4	11
Wuhan infected	Alpha	10	12	5
Wuhan infected	BA.1	10	13	5
Wuhan infected	BA.2	10	13	5
Wuhan infected	BA.5	10	0	5
Wuhan infected	Beta	10	13	5
Wuhan infected	Delta	10	12	5
Wuhan infected	Wuhan	10	13	5
Wuhan vaccinated (two doses)	Alpha	11	26	6
Wuhan vaccinated (two doses)	BA.1	11	13	6
Wuhan vaccinated (two doses)	BA.2	11	0	6
Wuhan vaccinated (two doses)	BA.5	11	4	6
Wuhan vaccinated (two doses)	Beta	11	28	6
Wuhan vaccinated (two doses)	Delta	11	26	6
Wuhan vaccinated (two doses)	Wuhan	11	30	6
Alpha infected	Alpha	9	13	6
Alpha infected	BA.1	9	13	6
Alpha infected	BA.2	9	13	6
Alpha infected	BA.5	9	0	6
Alpha infected	Beta	9	13	6
Alpha infected	Delta	9	13	6
Alpha infected	Wuhan	9	13	6

Beta infected	Alpha	6	15	12
Beta infected	BA.1	6	15	12
Beta infected	BA.2	6	0	12
Beta infected	BA.5	6	0	12
Beta infected	Beta	6	15	12
Beta infected	Delta	6	15	12
Beta infected	Wuhan	6	15	12
Delta infected	Alpha	5	15	16
Delta infected	BA.1	5	21	16
Delta infected	BA.2	5	11	16
Delta infected	BA.5	5	6	16
Delta infected	Beta	5	21	16
Delta infected	Delta	5	21	16
Delta infected	Wuhan	5	21	16

Table S6. Significant differences between NHP and human titers after adjusting for modelled effects, related to Figure 4. Log2 titers after adjusting for serum, assay and organism effects were tested for normality with a Shapiro-Wilk test. Many titers differed significantly from a normal distribution and a Wilcox rank sum test was used to compare human and NHP titers per serum group and variant. Significant ($p < 0.05$) results are shown. Calculations were performed using the rstatix package (v 0.7.2)[6].

Serum group	Variant	group1	group2	n1	n2	statistic	p-value	Significance level
Wuhan infected	Beta	Human	NHP	23	5	6	0.00059	***
Wuhan infected	Delta	Human	NHP	22	5	100	0.0028	**
Wuhan vaccinated (two doses)	BA.1	Human	NHP	24	6	30	0.0286	*
Wuhan vaccinated (two doses)	Delta	Human	NHP	37	6	170	0.038	*
Wuhan vaccinated (two doses)	Wuhan	Human	NHP	41	6	58	0.0374	*
alpha infected	BA.1	Human	NHP	22	6	28	0.0331	*
beta infected	BA.1	Human	NHP	21	12	56	0.00785	**
beta infected	Beta	Human	NHP	21	12	232	1.46e-05	****
beta infected	Delta	Human	NHP	21	12	72	0.0441	*
delta infected	Alpha	Human	NHP	20	16	34	1.65e-05	****
delta infected	BA.5	Human	NHP	11	16	131	0.0343	*
delta infected	Beta	Human	NHP	26	16	64	8.96e-05	****
delta infected	Delta	Human	NHP	26	16	365	1.41e-05	****
BA.1 infected	Alpha	Human	NHP	15	11	33	0.00915	**
BA.1 infected	Beta	Human	NHP	18	11	54	0.0442	*

References

[S1] Wilks, S.H. (2022). titertools: A statistical toolkit for the annalysis of censored titration data.

[S2] Wilks, S.H. (2022). Racmacs: R Antigenic Cartography Macros.

[S3] Rössler, A., Netzl, A., Knabl, L., Bante, D., Wilks, S.H., Borena, W., von Laer, D., Smith, D.J., and Kimpel, J. (2023). Characterizing SARS-CoV-2 neutralization profiles after bivalent boosting using antigenic cartography. Nat Commun 14, 5224, https://doi.org/10.1038/s41467-023-41049-4.

[S4] Wilks, S.H., Mühlemann, B., Shen, X., Türeli, S., LeGresley, E.B., Netzl, A., Caniza, M.A., Chacaltana-Huarcaya, J.N., Corman, V.M., Daniell, X. et al. (2023). Mapping SARS-CoV-2 antigenic relationships and serological responses. Science 382, eadj0070, https://doi.org/10.1126/science.adj0070.

[S5] Khare, S., Gurry, C., Freitas, L., Schultz, M.B., Bach, G., Diallo, A., Akite, N., Ho, J., Lee, R.T., Yeo, W. et al. (2021). GISAID's Role in Pandemic Response. China CDC Wkly 3, 1049-1051, https://doi.org/10.46234/ccdcw2021.255.

[S6] Kassambara, A. (2023). rstatix: Pipe-Friendly Framework for Basic Statistical Tests.
