# Supplementary material for: Nonhuman primate antigenic cartography of SARS-CoV-2
Source: Cell Rep. 2025 Jan 3;44(1):115140. doi: 10.1016/j.celrep.2024.115140 (PMC11781863; doi:10.1016/j.celrep.2024.115140)
Supplement: Document S2. Article plus supplemental information [file mmc3.pdf]

Cell Reports

Article

Nonhuman primate antigenic cartography of SARS-CoV-2

Graphical abstract

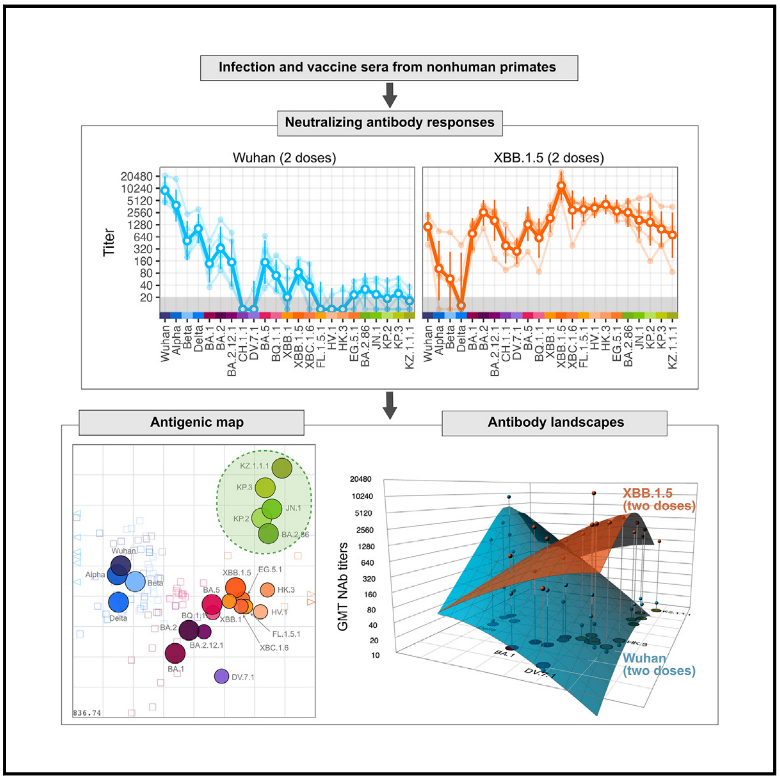

Authors

Annika Rössler, Antonia Netzl, Ninaad Lasrado, ..., Janine Kimpel, Derek J. Smith, Dan H. Barouch

Correspondence

dbarouch@bidmc.harvard.edu

In brief

Rössler et al. construct a nonhuman primate (NHP) antigenic map for SARS-CoV-2, showing the antigenic evolution and immune escape of BA.2.86 and descendants. By comparing matching human and NHP neutralization data and derived maps, they assess the suitability of NHPs as surrogate for missing human primary infection sera.

Highlights

- NHP antigenic map of SARS-CoV-2 variants shows escape of BA.2.86 and descended variants
- Recent variants BA.2.86, JN.1, KP.2, KP.3, and KZ.1.1.1 occupy a distant antigenic region
- Neutralization titers of NHPs and the derived antigenic map correspond well with human data
- NHP sera are suitable for the future antigenic characterization of SARS-CoV-2 variants

Check for updates

Rössler et al., 2025, Cell Reports *44*, 115140
January 28, 2025 © 2024 The Authors. Published by Elsevier Inc.
https://doi.org/10.1016/j.celrep.2024.115140

CellPress

Cell Reports

CellPress
OPEN ACCESS

Article

Nonhuman primate antigenic cartography of SARS-CoV-2

Annika Rössler,[1,6] Antonia Netzl,[2,6] Ninaad Lasrado,[1] Jayeshbhai Chaudhari,[1] Barbara Mühlemann,[3,4] Samuel H. Wilks,[2] Janine Kimpel,[5] Derek J. Smith,[2] and Dan H. Barouch[1,7,*]

[1]Center for Virology and Vaccine Research, Beth Israel Deaconess Medical Center, Harvard Medical School, Boston, MA 02215, USA

[2]Center for Pathogen Evolution, Department of Zoology, University of Cambridge, CB2 3EJ, Cambridge, Cambridgeshire, UK

[3]Institute of Virology, Charité - Universitätsmedizin Berlin, Corporate Member of Freie Universität Berlin, Humboldt Universität zu Berlin, and Berlin Institute of Health, 10117 Berlin, Berlin, Germany

[4]German Centre for Infection Research (DZIF), Partner Site Charité, 10117 Berlin, Berlin, Germany

[5]Institute of Virology, Department of Hygiene, Microbiology and Virology, Medical University of Innsbruck, Innsbruck, Tyrol 6020, Austria

[6]These authors contributed equally

[7]Lead contact

*Correspondence: dbarouch@bidmc.harvard.edu

https://doi.org/10.1016/j.celrep.2024.115140

SUMMARY

Virus neutralization profiles against primary infection sera and corresponding antigenic cartography are integral part of the COVID-19 and influenza vaccine strain selection processes. Human single variant exposure sera have previously defined the antigenic relationships among SARS-CoV-2 variants but are now largely unavailable due to widespread population immunity. Therefore, antigenic characterization of future SARS-CoV-2 variants will require an animal model, analogous to using ferrets for influenza virus. We evaluated neutralization profiles against 23 SARS-CoV-2 variants in nonhuman primates (NHPs) after single variant exposure and generated an NHP-derived antigenic map. We identified a distant antigenic region occupied by BA.2.86, JN.1, and the descendants KP.2, KP.3, and KZ.1.1.1. We also found that the monovalent XBB.1.5 mRNA vaccine induced broad immunity against the mapped antigenic space. In addition, substantial concordance was observed between our NHP-derived and two human antigenic maps, demonstrating the utility of NHPs as a surrogate for antigenic cartography in humans.

INTRODUCTION

The coronavirus disease 2019 (COVID-19) pandemic has entered an endemic phase. However, the severe acute respiratory syndrome coronavirus 2 (SARS-CoV-2) virus continues to evolve rapidly, and regular vaccine updates are necessary to maintain population immunity against emerging variants.[1–3] The composition and strain selection of future vaccines mainly depend on two factors: first, the antigenic properties of emerging variants relative to previous ones and consequently their potential immune escape and, second, the level of prevailing population immunity against these novel variants.[4] Neutralizing antibodies (NAbs) in cohorts reflecting the current state of population immunity help to evaluate the impact of emerging variants on existing immunity, but population immunity varies considerably on a global scale due to different exposure histories.[5–8] It is therefore important to understand the antigenic differences between previous and new SARS-CoV-2 variants, based on single exposure sera, without influence of pre-existing immunity.

Antigenic cartography is a computational tool that visualizes the antigenic relationships of virus variants on an antigenic map.[9] Ideally, neutralization titers in single variant exposure sera are used as input because samples after multiple exposures misrepresent antigenic relationships due to their high capacity for cross-variant neutralization.[10–12] Originally developed to study influenza A evolution, antigenic cartography has become an integral part of influenza vaccine strain selection over the past 20 years and has also been used to inform SARS-CoV-2 vaccine strain trials.[13–15] For SARS-CoV-2, antigenic maps derived from human single exposure sera are considered the gold standard. However, sera from humans exposed to only one variant are now rarely available for recent SARS-CoV-2 variants due to high levels of population immunity.[16] A recent study from our group suggests that the vast majority of individuals have natural immunity.[17] SARS-CoV-2 is thus approaching a similar level of endemicity as influenza. For influenza antigenic cartography, the lack of single human exposure sera for variant characterization is addressed by utilizing ferret animal models,[14,18] suggesting that suitable animal models will also be required moving forward for SARS-CoV-2.

Animal-derived antigenic maps of SARS-CoV-2 have to date only involved small animals, such as mice and hamsters, and are somewhat comparable with human data.[19–21] Given their genetic and physiological similarities to humans, nonhuman primates (NHPs) play a central role in translational medicine and vaccine development. They recapitulate many key immunologic features of humans following SARS-CoV-2 infection, thus serving as indispensable model in the COVID-19 research.[22–24] Although NHPs have been extensively used to characterize the

© 2024 The Authors. Published by Elsevier Inc.
This is an open access article under the CC BY license (http://creativecommons.org/licenses/by/4.0/).

A

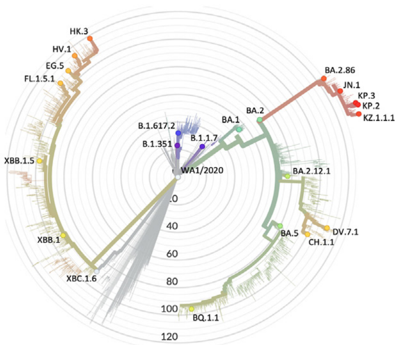

B

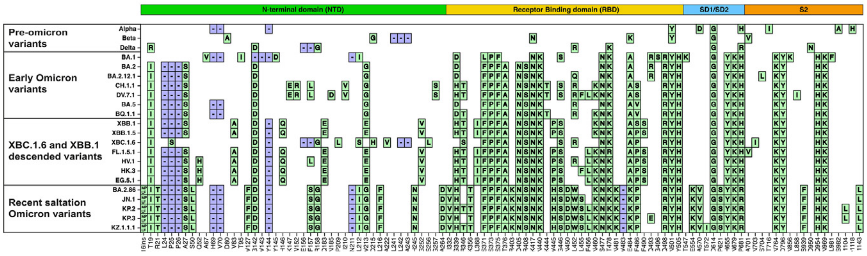

Figure 1. Panel of analyzed SARS-CoV-2 variants

(A) Evolutionary relationships of virus variants were visualized by a phylogenetic tree. Virus sequences were retrieved from the GISAID (Global Initiative on Sharing All Influenza Data) Initiative[33] (Table S1) and illustrated with clades.nextstrain.org.[34]

(B) Spike mutations of analyzed SARS-CoV-2 variants are shown relative to the ancestral Wuhan/WIV04/reference strain.[33] Amino acid substitutions of individual variants are indicated by green tiles and deletions by blue tiles. The figure was generated using a Jupyter Notebook from Jesse Bloom[35] and illustrated with Adobe Illustrator.

pathogenicity of SARS-CoV-2 and to study efficacy of vaccine candidates,[23–28] the antigenic properties of viral variants based on neutralization data in NHP sera and their correlation with human data have not yet been investigated.

In this study, we characterized the neutralization profiles against a diverse panel of 23 SARS-CoV-2 variants, including pre-Omicron (Wuhan, Alpha, Beta, and Delta), early Omicron (BA.1, BA.2, BA.2.12.1, CH.1.1, DV.7.1, BA.5, and BQ.1.1), XBC.1.6, and XBB.1-descended variants (XBB.1, XBB.1.5, FL.1.5.1, HV.1, HK.3, and EG.5), and recent saltation Omicron variants (BA.2.86, JN.1, KP.2, KP.3, and KZ.1.1.1) in sera collected from infected or vaccinated NHPs,[26,29–32] including animals immunized with a monovalent XBB.1.5 vaccine. Based on these NAb data, we generated the first NHP-derived antigenic map of SARS-CoV-2, elucidating the antigenic relationship of recent to previously circulating virus variants. Moreover, we analyzed the ability of a monovalent XBB.1.5 mRNA vaccine to confer immunity against the mapped antigenic space. To further assess whether NHPs resemble human NAb titer data, we performed a comparative analysis with two previously published human datasets, using a modified Bayesian framework that corrects for organism-, sera-, and assay-specific reactivities across species and compared antigenic maps. To the best of our knowledge, this is the first comparative analysis of NHP and human data on both NAb titer levels and antigenic properties of SARS-CoV-2 variants, presenting an important contribution to a broad research community.

RESULTS

NAb activity against antigenically diverse variants in NHP sera

We first evaluated NAb responses against a broad panel of 23 SARS-CoV-2 variants (Figure 1, Table S1) in our collection of NHP sera using a lentivirus-based pseudotype virus neutralization assay (Figure 2). The sera from 91 rhesus or cynomolgus macaques were collected as part of previous infection and vaccination studies[26,29–32] and were grouped according to SARS-CoV-2 exposure histories. All animals have been exposed to a single SARS-CoV-2 variant, either through infection ($n = 71$) with the Wuhan, Alpha, Beta, Gamma, Delta, Omicron BA.1, BA.2.12.1, BA.4, or BA.5 variant (Table S2) or by immunization ($n = 20$) with monovalent Wuhan, Beta, or XBB.1.5 vaccines (Table S3).

Animals infected with a pre-Omicron variant predominantly neutralized pre-Omicron variants at high titers, and conversely Omicron-infected cohorts showed higher NAb responses against Omicron variants (Figure 2A), which is in line with previous human and animal studies.[36–40] Infection or vaccination with Wuhan or Beta variants resulted in similar neutralization patterns but slightly more cross-neutralization in the Wuhan-infected than vaccinated cohorts. Vaccination with the XBB.1.5 variant resulted in broad cross-neutralization of most tested variants, except the Delta variant (Figure 2). NAb titers were highest against the homologous XBB.1.5 immunogen, followed by 2- to 4-fold lower titers against XBC.1.6, XBB.1 and its descendants, and BA.2 and further reduced titers against other variants.

We observed higher NAb titers for BA.2.86 than for JN.1 and its descendants KP.2, KP.3, and KZ.1.1.1 in sera from NHPs immunized with XBB.1.5 vaccines (Figure 2), while hardly any titers were detected in all other NHP cohorts. This pattern of sequentially decreasing NAb responses against these variants has also been observed in human multi-exposure sera[6,41–45] and is in line with viral evolution toward increased immune escape.

In general, we found considerable differences in titer magnitudes among the study cohorts based on vaccination or infection and time intervals between antigen exposure and blood collection (Tables S2 and S3). Two vaccine doses induced the highest NAb titers against the homologous antigen 14 days post-vaccination (Figure 2B). NAb titers in sera collected 26 days post-single dose vaccination (Wuhan, Beta) were of a similar magnitude to titers observed 20 days post-infection (Wuhan, Alpha) (Figure 2A). Most samples collected by day 10 post-infection had NAb titers

CellPress
OPEN ACCESS

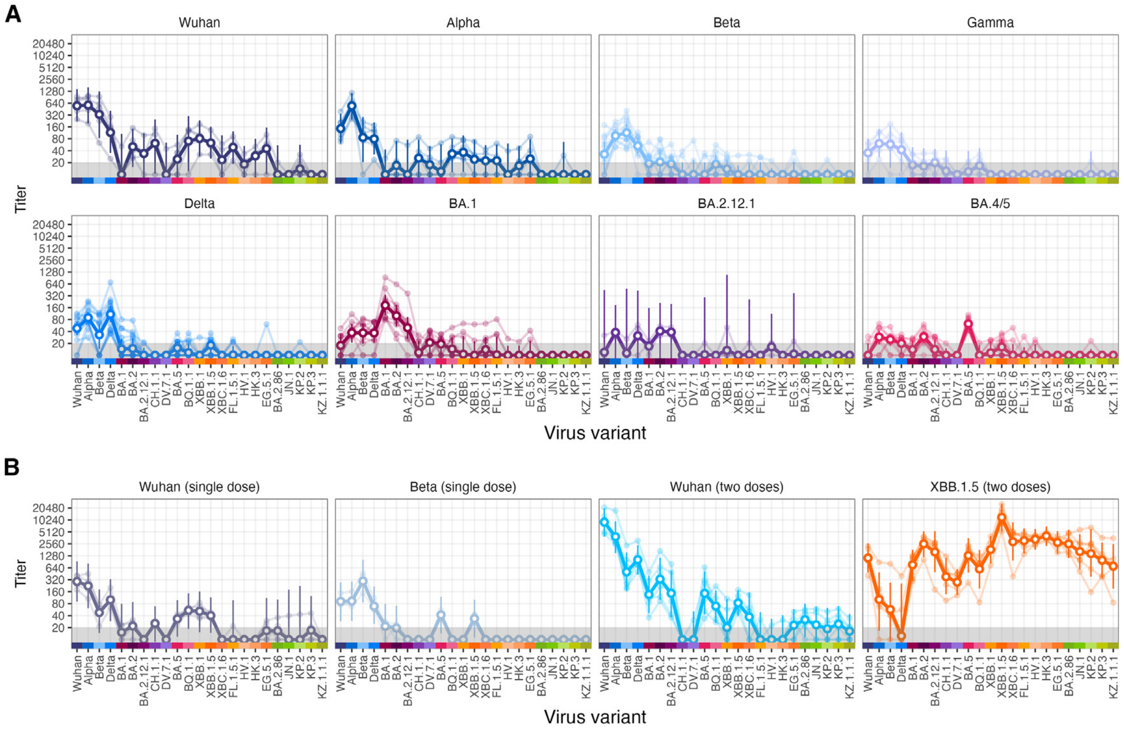

Figure 2. Neutralization of SARS-CoV-2 variants by NHPs with different exposure histories

(A) Sera were collected from unvaccinated animals after challenge with Wuhan (n = 5), Alpha (n = 6), Beta (n = 12), Gamma (n = 6), Delta (n = 16), Omicron BA.1 (n = 11), BA.2.12.1 (n = 3), or BA.4/BA.5 (n = 3/n = 9) variant and (B) from animals vaccinated with a single dose of adenoviral Wuhan (Ad26.CoV.2.S, n = 4) or Beta (Ad26.CoV.2.S.351, n = 4) vaccine, or two homologous doses of monovalent Wuhan (n = 6) or XBB.1.5 (n = 6) mRNA vaccine. NAb titers against indicated SARS-CoV-2 variants (x axis) were assessed using a luciferase-based pseudovirus neutralization assay. Each panel is labeled with the respective study cohort and colored according to the exposed antigen variant to match colors in the subsequent antigenic map. Faint thin lines show individual serum titers, while bold lines represent geometric mean titers (GMTs) with 95% confidence interval (CI) of each study cohort.

close to the limit of detection (LOD) against most variants, including the infecting variant (Figure 2A: Gamma, BA.2.12.1, BA.4/5). What appears to be considerable cross-neutralization in these early sampled sera could potentially be low, non-specific antibody measurements due to a not fully matured antibody response, or measurement noise close to the LOD.

Antigenic cartography reveals ongoing virus evolution in antigenically distinct regions

To characterize the antigenic relationship of analyzed SARS-CoV-2 variants, we next used our NHP dataset to perform antigenic cartography as previously described.[9,38] Briefly, virus variants are positioned relative to each other in a multi-dimensional space, where the Euclidean distances represent antigenic relationships. In 2D, this results in an antigenic map where map distances reflect NAb escape (Figure 3A). Antigenically similar virus variants are located close to each other, while antigenically different variants with low cross-neutralization are positioned further apart. We found higher than homologous titers against Alpha in many serum cohorts, indicating that the specific Alpha virus has high reactivity, and we optimized its reactivity as described in the Reactivity adjustments section in the STAR Methods (Figure S1).

We found that the CH.1.1 variant reduced map stability, resulting in a map with two distinct optimal conformations in 2D. In 3D, both conformations were present, and the 2D bistability is hence an effect of dimensionality reduction and downwards projection from 3D onto 2D (Figure S2). A map without CH.1.1 was robust to the exclusion of sera and antigens (Figures S3 and S4). Due to the increased stability and the potential of noise in low or non-detectable CH.1.1 titers in most serum cohorts, we focused our downstream analysis on an antigenic map excluding CH.1.1 (Figure 3).

In line with other animal and human antigenic maps,[19,21,37,46,47] pre-Omicron (Wuhan, Alpha, Beta, and Delta) variants, some early-Omicron (BA.1, BA.2, and BA.2.12.1) variants, recombinant variants (XBC.1.6 and XBB.1 descendants), and recent saltation Omicron variants (BA.2.86, JN.1, KP.2, KP.3, and KZ.1.1.1) grouped together and occupied distinct antigenic regions in our antigenic map (indicated by different color schemes in Figures 3A and S5). Reflecting their strong neutralization escape from earlier sera, the BA.2.86; JN.1; and subsequent KP.2, KP.3, and KZ.1.1.1 variants were found in a distant region in antigenic space, with BA.2.86 closest and KZ.1.1.1 furthest apart from previous recombinant variants. The Omicron BA.5 variant and its descendant BQ.1.1 were located close to the XBB.1 descendants; the BA.2.75 subvariant DV.7.1 was found at somewhat similar distance to initial Omicron variants (BA.1, BA.2), but more distant to XBB.1 descendants (Figure 3A). A 3D map exhibited largely the same properties as the 2D map but showed BQ.1.1 and DV.7.1 more distinct from early Omicron variants (Figure S6).

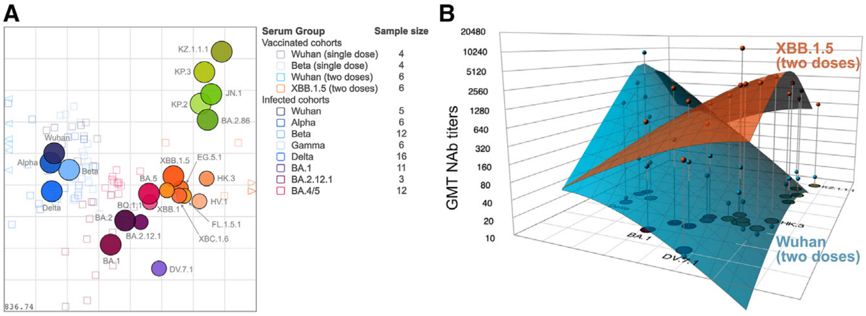

Figure 3. Antigenic cartography of SARS-CoV-2 variants and antibody landscapes following monovalent Wuhan or XBB.1.5 vaccination

(A) An antigenic map of SARS-CoV-2 variants was constructed as previously described[9,38] from NHP sera following single variant exposure by infection or vaccination. Analyzed virus variants are denoted as colored circles and individual sera are displayed by open squares in colors corresponding to their exposed antigen. Major variants are shown as bigger circles and minor variants as smaller circles. Sera located outside of the represented area are indicated by triangles. A non-zoomed map is shown in Figure S5. All virus variants are positioned relative to each other (*x* and *y* axis orientation is relative) with one grid distance within this map corresponding to one 2-fold dilution of neutralization titers. The legend shows study cohorts and sample sizes used for the construction of the antigenic map. The small number in the bottom left indicates the map stress, i.e., the error between titer measurement and map position.

(B) Neutralization profiles of NHPs after two doses of monovalent Wuhan (blue) or XBB.1.5 (orange) mRNA vaccines were visualized in 3D above the antigenic map by GMT antibody landscapes. Measured GMTs against each variant are indicated as colored points above the respective variant; the surface shows the fitted antibody landscape. The z axis reflects NAb titers, with every 4-fold increase from titer 10 marked.

Variation in a variant's position indicates poor triangulation due to few titers above the limit of detection (>LOD. We found that overall low titers at sampling time points early post-infection condensed the map and poorly resolved the position of variants later than BQ.1.1 (Figures S7A–S7C). Samples from later time points with higher NAb titers and a broader range of >LOD titers improved positional resolution and distinction of variants (Figures S7D–S7F). In general, the resolution of pre-Omicron variants, for which most sera had >LOD titers, was better than for BA.2.75 and subsequent variants (Figure S8B), whose positions were more sensitive to the exclusion of individual measurements and measurement errors (Figures S8C–S8E). We expect further homologous sera raised against saltation omicron variants to overcome this limitation (Figure S7B) shows the impact of removing sera with >LOD titers on BA.2.75, BA.2.86, and descendants).

Since it is important that available vaccines protect against circulating variants, we further evaluated the magnitude and breadth elicited by different COVID-19 vaccine compositions. Using our antigenic map as the base plane, we visualized and compared the neutralization profile of two-dose Wuhan- or XBB.1.5-immunized NHPs by antibody landscapes (Figure 3B). The magnitudes of these antibody landscapes reflect their neutralization capacity against underlying variants. We found complementary antibody landscapes in Wuhan- and XBB.1.5-vaccinated cohorts. While vaccination with the Wuhan strain gives immunity to all pre-Omicron variants, we observed a steep decline against the antigenic space of subsequent variants. Conversely, the monovalent XBB.1.5 vaccine primarily induced immunity to virus variants concentrated around the cluster of XBB.1-descended variants, demonstrating the ability to generate *de novo* immune responses against these variants in the absence of prior immunity, but lower responses against more distant antigenic spaces.

NAb responses induced in NHPs correspond well with human single variant exposure sera

To evaluate the suitability of NHP sera as surrogate for human sera, we compared the NHP neutralization profiles with those of matching human study cohorts from two previously published datasets[36,46] (Figure 4; Tables S4 and S5). Since the data of human study cohorts were assessed using an authentic virus in Vero cells overexpressing TMPRSS2 and ACE2 or lentiviral pseudotype assay in 293T cells expressing ACE2 cells, as done for the NHP data, we evaluated not only NAb titer levels but also the impact of different neutralization assay types. Across all serum groups, we found similar neutralization patterns in NHPs and humans. To further control for and quantify titer magnitude differences based on specific assay, organism, and individual serum reactivities, we adapted a previously described Bayesian framework[19,20] that was developed to compare neutralization titers from different sources (laboratories and organisms). Because both lentiviral assays and the live virus assay were performed in different cell types, the model could not distinguish between effects caused by virus or cell type and we refer for any assay-caused effect to the virus type.

After adjusting for the aforementioned effects on titers, titers from all three datasets corresponded very well (Figure 4). Although there were still significant differences between human and NHP titers for individual variants in specific cohorts (Table S6), no overall difference between titers from human and NHPs was detected (1.06-fold higher NHP reactivity, not-significant) (Figure S9). Moreover, the impact of authentic vs. pseudovirus assay was estimated to be larger and more significant than the difference between organisms, with lentiviral pseudotype titers being on average 1.6-fold higher than authentic virus titer measurements (Figure S9). This demonstrates that NHP titers substantially reflect human NAb responses, with same or better correlation of NHP data with human data than the two human datasets with each other after adjusting for described effects (Figures S9 and S10).

Similar conformation of NHP and human antigenic maps

Finally, a comparison of the NHP-derived to human antigenic maps was performed, which also revealed good correspondence on the antigenic level (Figure 5). We observed a comparable clustering of analyzed virus variants, and relative positions of

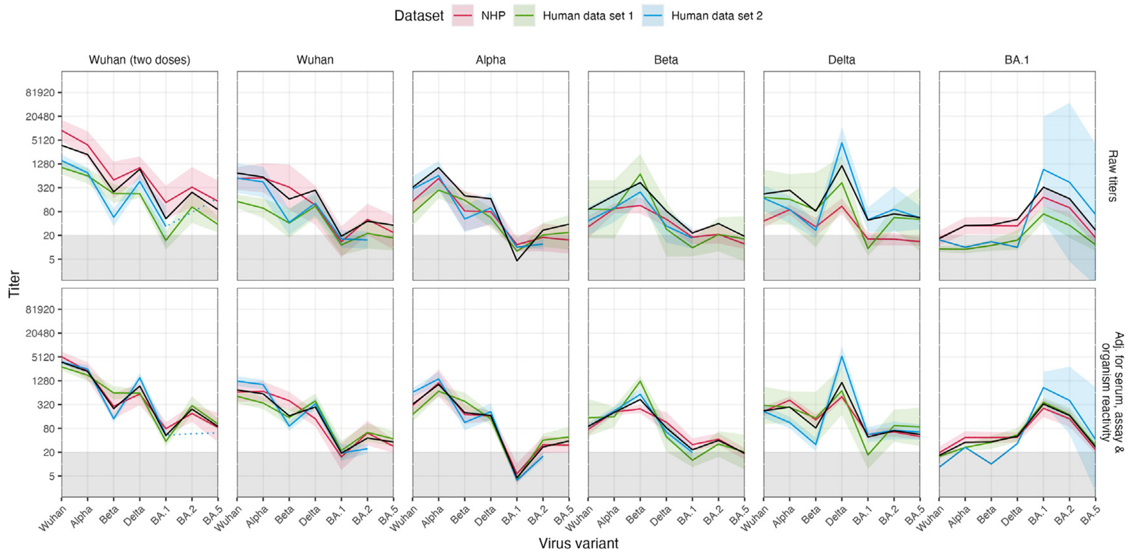

Figure 4. Neutralizing antibody responses of matched NHP and human cohorts

NAb responses in NHP sera against Wuhan, Alpha, Beta, Delta, and Omicron BA.1, BA.2, and BA.5 variants were compared with two previously published human datasets (Tables S4 and S5). Neutralization titers in human samples were assessed using live virus in Vero-TMPRSS2/ACE2 cells (human dataset 1, Rössler et al.[46]) and lentivirus neutralization assays in 293T/ACE2, same as NHP data (human dataset 2, Wilks et al.[36]). To control titer variations due to different reactivities of individual sera and estimate assay- and species-specific effects, titers were adjusted using a Bayesian framework.[19,20] The columns show NHP (red), human(green and blue), and, in black, the derived combined GMT (geometric mean titers) ± 95% CI (confidence interval) as bold colored line and shaded area for each serum group. The black line represents the estimated GMT per serum group across organisms after adjusting for serum, assay, and organism effects. The upper row shows GMTs of raw titers with titers <LOD (limit of detection ≤20) set to LOD/2, while the lower row shows GMTs ± 95% CI after adjusting for estimated serum, assay, and organism reactivity differences (Figure S8).

individual variants showed good agreement, with the biggest differences for BA.1 and BA.2. They can partly be explained by the different sera compositions used for the calculation of the antigenic maps (Table S5, Wilks et al., and Rössler et al.[36,46]) and map sensitivity to the inclusion of homologous sera. While human BA.2 sera had a strong influence on BA.1 and BA.2 positions in human maps,[36,46] corresponding NHP sera were not available. The two human maps show a larger spread of the variants, while low titers against most variants and seemingly broad cross-neutralization in NHP sera sampled early post-infection as discussed earlier resulted in a condensation of the virus variants in the center of the map (Figure S7). Antigenic distances reflect neutralization titer fold changes to exposed antigens; consequently, lower NAb titers against the homologous antigen result in reduced antigenic distances.

DISCUSSION

Future antigenic characterization of emerging SARS-CoV-2 variants will require appropriate animal models, since human single exposure sera are practically no longer available due to high population immunity.[16,17] Antigenic cartography forms an integral part of influenza vaccine strain selection and utilizes ferret sera as a substitute for human single exposure sera given a widely exposed population.[13,14,18] The assessment of antigenic differences among SARS-CoV-2 variants with the availability of primary infection human sera is only starting to be formalized, and the suitability of animal models will be a determining factor in the SARS-CoV-2 vaccine variant selection process. Given their high genetic and physiological similarities with humans, NHPs represent a useful animal model. To the best of our knowledge, this study represents the first comprehensive comparison of NHP and human datasets for antigenic properties of SARS-CoV-2 variants. These findings may also be applicable for other viruses.

Our modified modeling framework[19,20] revealed that NHPs substantially resemble humans in their NAb responses after SARS-CoV-2 infection and vaccination (Figure 4). Differences in titers due to different assay types (authentic virus in Vero-TMPRSS2/ACE2 vs. lentiviral pseudotype assay in 293T/ACE2) were larger than those due to species (NHP vs. human) (Figure S9). As recently seen for hamster sera,[20] NHP sera could serve as substitute for human NAb responses in the absence of specific human sera, such as single exposure sera against emerging SARS-CoV-2 variants. A considerable advantage of NHPs over small animal models is the amount of serum obtainable per animal. Routine antigenic characterizations for influenza vaccine strain selection involve titrating a single ferret serum over a large number of strains.[48] With the continuous emergence of new SARS-CoV-2 variants, the number of strains to be tested will continue to increase, posing a challenge for the lower serum volume obtainable from rodents. Moreover, the B cell repertoire of NHPs is by nature more similar to that of humans than that of hamster, and NHPs have orthologs for the germline genes that are frequently used for generating NAb responses against SARS-CoV-2 in humans.[49–53]

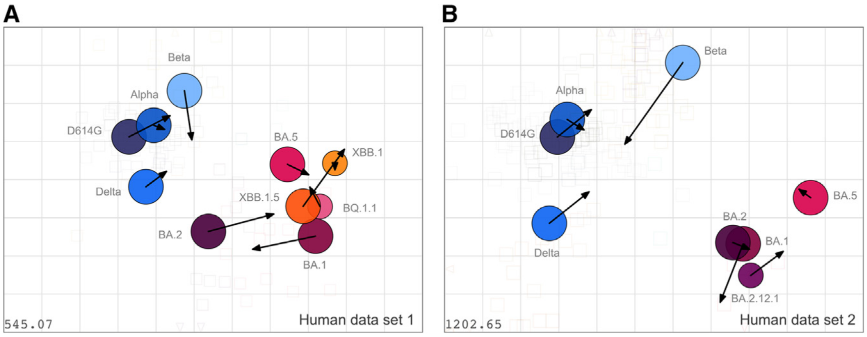

Figure 5. Comparison of NHP-derived antigenic map with two previously published human antigenic maps

Arrows point to the variants' positions in the NHP map (Figure 3) from (A) the previously published human dataset 1 map[46] and (B) the human dataset 2 map.[36] Virus variants are shown by colored circles, and the colors correspond to the colors used in Figure 3.

In the antigenic map of our panel of SARS-CoV-2 variants as inferred by NHP neutralization data, we found virus clusters of pre-Omicron, early-Omicron, and recombinant (XBC.1.6, XBB.1, and XBB.1 descendants) variants in antigenically distinct regions, which is in good concordance with two human antigenic maps (Figure 5). As first published by Van der Straten et al.,[47] numerous antigenic maps show pre-Omicron and Omicron variants in separate clusters.[11,19,36,37,46] To the best of our knowledge, the presented map is the first to include JN.1 and its subvariants KP.2, KP.3, and KZ.1.1.1, showing their increased immune escape from earlier sera compared to BA.2.86 and JN.1. A recent mouse-derived map located BA.2.86 antigenically distinct from both ancestral and recombinant variants,[54] which is in line with our data. By contrast, in another antigenic map constructed from hamster primary infection sera, JN.1 is co-located with BA.5, XBB.1.5, and recombinant descendants.[21] Although JN.1's distance to XBB.1.5 is similar to the distance in our map in antigenic distance units, its relative escape from hamster sera is much less than that in our NHP map. Considering the enhanced escape of BA.2.86, JN.1, and its subvariants in humans following XBB.1.5 vaccination or breakthrough infection,[6,41–44] and their reduced sensitivity to widely used monoclonal antibody (mAb) sotrovimab and various class 1 mAbs compared to XBB lineages,[6,55] we would expect these characteristics to be reflected by larger antigenic distances, as seen in our data and the mouse map.

While there is overall large agreement between map conformations, contributors to different map conformations are the panel of analyzed sera as well as varying time point of sampling as, e.g., low titers in NHP samples taken up to 10 days post-infection resulted in low map distances from the homologous variant to <LOD variants and a contraction of the map (Figures 5 and S7). Early samples might not resemble a fully mature NAb response and therefore underestimate the real antigenic distances. Thus, for future antigenic studies in NHPs, we ideally recommend using samples collected 3–4 weeks post-challenge. Additional homologous sera, e.g., raised against recent variants, could help to refine the resolution of BA.2.87, JN.1, and its descendants positions relative to each other and to previously circulating variants.

The current XBB.1.5 vaccine elicited a broad *de novo* NAb response against homologous and closely related antigens at high magnitudes in NHPs, but also against more distant variants, such as BA.2.86 and subsequent variants, and surprisingly ancestral variants albeit at lower titers. This suggests that XBB.1.5 is a potent immunogen inducing broad cross-reactive NAb responses in naive individuals. In humans with prior immunity against historical variants, the XBB.1.5 booster dose elicited immune responses against recent recombinant variants, but the recall of pre-existing immunity dominated the overall NAb response.[8,41,56] This was also evident in NHPs primed with historic vaccines after protein-based XBB.1.5 booster dose.[57] Previous mouse and human studies showed improved cross-neutralization after XBB.1.5 vaccination only by shared spike epitopes but no *de novo* XBB.1.5-specific responses.[41,56] Similar immune imprinting effects might occur with the JN.1- and KP.2-specific vaccine updates as recently recommended by the World Health Organization (WHO) and U.S. Food and Drug Administration (FDA), respectively.[3,58] Although the JN.1 and KP.2 vaccine updates might help to mitigate the 1- to 5-fold lower titers against JN.1 compared to XBB lineages in humans,[41,56] overcoming the interference with prior immunity and generating specific NAb responses against new areas of antigenic space will remain a key challenge in COVID-19 vaccination.

In our map, constructed using a diverse serum panel, BA.2.86; JN.1; and the descendants KP.2, KP.3, and KZ.1.1.1 escape neutralization in single variant exposure sera and move into a new region of antigenic space, which is in line with their enhanced immune escape from human multi-exposure sera.[43,44] Transitions of antigenic clusters are a natural process of viral antigenic evolution and lead to regular strain updates of influenza vaccines.[13,14,48] In this light, the recent recommendations by the WHO and the FDA to include JN.1 or KP.2,[3,58] respectively, in the upcoming COVID-19 vaccine update seem reasonable to ensure optimal protection against this new region of antigenic space. First NAb data in humans indeed showed an improved neutralization of JN.1 and subsequent variants after JN.1 booster dose.[59]

The harmonization and comparability of datasets derived from different species is an important issue to bridge human and animal data and for strain selection of COVID-19 vaccine updates.[19] NHPs represent a unique animal model for studying antibody responses following SARS-CoV-2 exposure due to their close genetic relationship to humans and similar B cell receptor repertoire. However, the ethical use of NHP sera for antigenic cartography is essential. In this study, only existing sera from previous studies have been utilized and no additional animals have been infected or vaccinated to generate present data. Moving forward, sera raised against future emerging variants might be needed to define their antigenic relationship to

CellPress
OPEN ACCESS

prior variants accurately. Given the high costs and ethical considerations associated with NHP research, we recommend that future antigenic characterization of SARS-CoV-2 variants in NHPs utilize sera collected as part of other challenge or immunization studies when possible, and new samples should only be generated if essential gaps in knowledge exist that cannot be addressed using small animal models.

Our data suggest that NHPs are a valuable contribution to the future antigenic characterization of SARS-CoV-2 in three ways: first, we show that NAb responses in NHPs are generally comparable to human responses; second, the amount of serum obtainable from NHPs is far greater than the amount of serum obtainable from rodents; and third, the immunological properties of NHPs are similar to those of humans, which might also be beneficial to study complex exposure histories, mucosal immunity, and phenomena such as immune imprinting, for which small animal models might be less suitable. Our data suggest that NHPs are a useful surrogate for humans for assessing antigenic differences and for antigenic cartography and should help inform strain selection of future COVID-19 vaccines.

Limitations of the study

The data used in this study were not generated for this purpose and therefore come with limitations. Serum samples from NHPs taken early (up to ten days) post-infection might not capture a fully matured NAb response, and seemingly broad cross-neutralization might be due to generally low titers. Moreover, NHP sera raised against more recent virus variants (BA.2.86 and later) were not available, which would improve antigenic map resolution. Additionally, no data from matching human study cohorts were available for variants beyond BA.1 for a direct comparison with our NHP data. Finally, the neutralization data were derived from assays using different cell types, highlighting the need of further studies to quantify the impact of cell type variations on NAb magnitudes.

RESOURCE AVAILABILITY

Lead contact

Further information and requests for resources and reagents should be directed to the lead contact Dan H. Barouch (dbarouch@bidmc.harvard.edu).

Materials availability

This study did not generate new unique reagents.

Data and code availability

- All data shown in this study have been deposited at Zenodo: https://doi.org/10.5281/zenodo.12732108 and are publicly available. The human data used in this study were originally published in Wilks et al.[36,60] and Rössler et al.[46,61] and can be accessed at the associated repositories at Zenodo: https://doi.org/10.5281/zenodo.8195839 and Zenodo: https://doi.org/10.5281/zenodo.8199367, respectively.
- All original code is deposited at Zenodo: https://doi.org/10.5281/zenodo.12732108 and is publicly available.
- Any additional information required to reanalyze the data reported in this paper is available from the lead contact upon request.

ACKNOWLEDGMENTS

We thank Nicole Hachmann, Darren Ty, Osceola Heard, and Reed Boduch for their excellent technical support. This work was supported by the NIH grant CA260476 (D.H.B.), NIH contract 75N93021C00014 SAVE program (A.R., A.N., N.L., J.K., D.J.S., and D.H.B.), Gates Foundation grant INV-042469 (D.H.B.), and the Gates Cambridge Trust (A.N.).

AUTHOR CONTRIBUTIONS

A.R. and A.N. contributed equally to this work. A.R. initiated the study and performed the neutralization assays. A.N. performed the antigenic cartography and bioinformatical analysis. A.R. and A.N. generated the figures and co-wrote the original draft of the manuscript. N.L. and J.C. helped with the pseudovirus generation and assisted with the neutralization assays. S.H.W. and B.M. contributed to the code. S.H.W. and J.K. provided the human datasets. D.J.S. supervised the antigenic cartography. D.H.B. conceptualized and supervised the study and edited the manuscript. All co-authors contributed to the review and editing of the final manuscript prior to submission.

DECLARATION OF INTERESTS

J.K. is listed as an inventor on patents related to vesicular stomatitis virus-based oncolytic viruses.

STAR★METHODS

Detailed methods are provided in the online version of this paper and include the following:

- KEY RESOURCES TABLE
- EXPERIMENTAL MODEL AND STUDY PARTICIPANT DETAILS
  - Animals
  - Cell lines
- METHOD DETAILS
  - Pseudovirus neutralization assay
  - Antigenic cartography
  - Reactivity adjustments
  - Titer modeling
- QUANTIFICATION AND STATISTICAL ANALYSIS

SUPPLEMENTAL INFORMATION

Supplemental information can be found online at https://doi.org/10.1016/j.celrep.2024.115140.

Received: August 24, 2024
Revised: November 4, 2024
Accepted: December 11, 2024
Published: January 3, 2025

REFERENCES

1. W.H.O. (2022). Technical Advisory Group on COVID-19 Vaccine Composition (TAG-CO-VAC). Interim statement on the composition of current COVID-19 vaccines. https://www.who.int/news/item/17-06-2022-interim-statement-on–the-composition-of-current-COVID-19-vaccines
2. W.H.O. (2023). Technical Advisory Group on COVID-19 Vaccine Composition (TAG-CO-VAC). Statement on the antigen composition of COVID-19 vaccines. https://www.who.int/news/item/26-04-2024-statement-on-the-antigen-composition-of-covid-19-vaccines
3. W.H.O. (2024). Technical Advisory Group on COVID-19 Vaccine Composition (TAG-CO-VAC). Statement on the antigen composition of COVID-19 vaccines. https://www.who.int/news/item/26-04-2024-statement-on-the-antigen-composition-of-covid-19-vaccines.
4. DeGrace, M.M., Ghedin, E., Frieman, M.B., Krammer, F., Grifoni, A., Alisoltani, A., Alter, G., Amara, R.R., Baric, R.S., Barouch, D.H., et al. (2022). Defining the risk of SARS-CoV-2 variants on immune protection. Nature *605*, 640–652. https://doi.org/10.1038/s41586-022-04690-5.

5. Lassaunière, R., Polacek, C., Utko, M., Sørensen, K.M., Baig, S., Ellegaard, K., Escobar-Herrera, L.A., Fomsgaard, A., Spiess, K., Gunalan, V., et al. (2023). Virus isolation and neutralisation of SARS-CoV-2 variants BA.2.86 and EG.5.1. Lancet Infect. Dis. *23*, e509–e510. https://doi.org/10.1016/s1473-3099(23)00682-5.
6. Yang, S., Yu, Y., Xu, Y., Jian, F., Song, W., Yisimayi, A., Wang, P., Wang, J., Liu, J., Yu, L., et al. (2024). Fast evolution of SARS-CoV-2 BA.2.86 to JN.1 under heavy immune pressure. Lancet Infect. Dis. *24*, e70–e72. https://doi.org/10.1016/s1473-3099(23)00744-2.
7. Willett, B.J., Logan, N., Scott, S., Davis, C., McSorley, T., Asamaphan, P., Hosie, M.J., Olmo, P., Grove, J., Orton, R., et al. (2023). Omicron BA.2.86 cross-neutralising activity in community sera from the UK. Lancet *402*, 2075–2076. https://doi.org/10.1016/s0140-6736(23)02397-8.
8. Lasrado, N., Rössler, A., Rowe, M., Collier, A.R.Y., and Barouch, D.H. (2024). Neutralization of SARS-CoV-2 Omicron subvariant BA.2.87.1. Vaccine *42*, 2117–2121. https://doi.org/10.1016/j.vaccine.2024.03.007.
9. Smith, D.J., Lapedes, A.S., de Jong, J.C., Bestebroer, T.M., Rimmelzwaan, G.F., Osterhaus, A.D.M.E., and Fouchier, R.A.M. (2004). Mapping the antigenic and genetic evolution of influenza virus. Science *305*, 371–376. https://doi.org/10.1126/science.1097211.
10. Alves, K., Plested, J.S., Galbiati, S., Chau, G., Cloney-Clark, S., Zhu, M., Kalkeri, R., Patel, N., Smith, K., Marcheschi, A., et al. (2023). Immunogenicity of a Fourth Homologous Dose of NVX-CoV2373. N. Engl. J. Med. *388*, 857–859. https://doi.org/10.1056/NEJMc2215509.
11. Wang, W., Lusvarghi, S., Subramanian, R., Epsi, N.J., Wang, R., Goguet, E., Fries, A.C., Echegaray, F., Vassell, R., Coggins, S.A., et al. (2022). Antigenic cartography of well-characterized human sera shows SARS-CoV-2 neutralization differences based on infection and vaccination history. Cell Host Microbe *30*, 1745–1758.e7. https://doi.org/10.1016/j.chom.2022.10.012.
12. Mykytyn, A.Z., Fouchier, R.A., and Haagmans, B.L. (2023). Antigenic evolution of SARS coronavirus 2. Curr. Opin. Virol. *62*, 101349. https://doi.org/10.1016/j.coviro.2023.101349.
13. Fouchier, R.A.M., and Smith, D.J. (2010). Use of antigenic cartography in vaccine seed strain selection. Avian Dis. *54*, 220–223. https://doi.org/10.1637/8740-032509-ResNote.1.
14. Fonville, J.M., Fraaij, P.L.A., de Mutsert, G., Wilks, S.H., van Beek, R., Fouchier, R.A.M., and Rimmelzwaan, G.F. (2016). Antigenic Maps of Influenza A(H3N2) Produced With Human Antisera Obtained After Primary Infection. J. Infect. Dis. *213*, 31–38. https://doi.org/10.1093/infdis/jiv367.
15. Branche, A.R., Rouphael, N.G., Diemert, D.J., Falsey, A.R., Losada, C., Baden, L.R., Frey, S.E., Whitaker, J.A., Little, S.J., Anderson, E.J., et al. (2023). Comparison of bivalent and monovalent SARS-CoV-2 variant vaccines: the phase 2 randomized open-label COVAIL trial. Nat. Med. *29*, 2334–2346. https://doi.org/10.1038/s41591-023-02503-4.
16. Jones, J.M., Manrique, I.M., Stone, M.S., Grebe, E., Saa, P., Germanio, C.D., Spencer, B.R., Notari, E., Bravo, M., Lanteri, M.C., et al. (2023). Estimates of SARS-CoV-2 Seroprevalence and Incidence of Primary SARS-CoV-2 Infections Among Blood Donors, by COVID-19 Vaccination Status - United States, April 2021-September 2022. MMWR Morb. Mortal. Wkly. Rep. *72*, 601–605. https://doi.org/10.15585/mmwr.mm7222a3.
17. Nkolola, J.P., Liu, J., Collier, A.R.Y., Jacob-Dolan, C., Senussi, Y., Borberg, E., Swank, Z., Walt, D.R., and Barouch, D.H. (2024). High Frequency of Prior SARS-CoV-2 Infection by Sensitive Nucleocapsid Assays. J. Infect. Dis. *230*, e601–e604. https://doi.org/10.1093/infdis/jiae174.
18. Maher, J.A., and DeStefano, J. (2004). The Ferret: An Animal Model to Study Influenza Virus. Lab Anim. *33*, 50–53. https://doi.org/10.1038/laban1004-50.
19. Mühlemann, B., Wilks, S.H., Baracco, L., Bekliz, M., Carreño, J.M., Corman, V.M., Davis-Gardner, M.E., Dejnirattisai, W., Diamond, M.S., Douek, D.C., et al. (2024). Comparative analysis of SARS-CoV-2 neutralization titers reveals consistency between human and animal model serum and across assays. Sci. Transl. Med. *16*, eadl1722. https://doi.org/10.1126/scitranslmed.adl1722.
20. Rössler, A., Netzl, A., Knabl, L., Wilks, S.H., Mühlemann, B., Türeli, S., Mykytyn, A., von Laer, D., Haagmans, B.L., Smith, D.J., and Kimpel, J. (2024). Direct comparison of SARS-CoV-2 variant specific neutralizing antibodies in human and hamster sera. npj Vaccines *9*, 85. https://doi.org/10.1038/s41541-024-00888-y.
21. Wang, W., Bhushan, G., Paz, S., Stauft, C.B., Selvaraj, P., Goguet, E., Bishop-Lilly, K.A., Subramanian, R., Vassell, R., Lusvarghi, S., et al. (2024). Human and hamster sera correlate well in identifying antigenic drift among SARS-CoV-2 variants, including JN.1. J. Virol. *98*, e0094824. https://doi.org/10.1128/jvi.00948-24.
22. Lu, S., Zhao, Y., Yu, W., Yang, Y., Gao, J., Wang, J., Kuang, D., Yang, M., Yang, J., Ma, C., et al. (2020). Comparison of nonhuman primates identified the suitable model for COVID-19. Signal Transduct. Target. Ther. *5*, 157. https://doi.org/10.1038/s41392-020-00269-6.
23. Munster, V.J., Feldmann, F., Williamson, B.N., van Doremalen, N., Pérez-Pérez, L., Schulz, J., Meade-White, K., Okumura, A., Callison, J., Brumbaugh, B., et al. (2020). Respiratory disease in rhesus macaques inoculated with SARS-CoV-2. Nature *585*, 268–272. https://doi.org/10.1038/s41586-020-2324-7.
24. Shan, C., Yao, Y.-F., Yang, X.-L., Zhou, Y.-W., Gao, G., Peng, Y., Yang, L., Hu, X., Xiong, J., Jiang, R.-D., et al. (2020). Infection with novel coronavirus (SARS-CoV-2) causes pneumonia in Rhesus macaques. Cell Res. *30*, 670–677. https://doi.org/10.1038/s41422-020-0364-z.
25. van Doremalen, N., Lambe, T., Spencer, A., Belij-Rammerstorfer, S., Purushotham, J.N., Port, J.R., Avanzato, V.A., Bushmaker, T., Flaxman, A., Ulaszewska, M., et al. (2020). ChAdOx1 nCoV-19 vaccine prevents SARS-CoV-2 pneumonia in rhesus macaques. Nature *586*, 578–582. https://doi.org/10.1038/s41586-020-2608-y.
26. Yu, J., Tostanoski, L.H., Peter, L., Mercado, N.B., McMahan, K., Mahrokhian, S.H., Nkolola, J.P., Liu, J., Li, Z., Chandrashekar, A., et al. (2020). DNA vaccine protection against SARS-CoV-2 in rhesus macaques. Science *369*, 806–811. https://doi.org/10.1126/science.abc6284.
27. Mercado, N.B., Zahn, R., Wegmann, F., Loos, C., Chandrashekar, A., Yu, J., Liu, J., Peter, L., McMahan, K., Tostanoski, L.H., et al. (2020). Single-shot Ad26 vaccine protects against SARS-CoV-2 in rhesus macaques. Nature *586*, 583–588. https://doi.org/10.1038/s41586-020-2607-z.
28. Corbett, K.S., Flynn, B., Foulds, K.E., Francica, J.R., Boyoglu-Barnum, S., Werner, A.P., Flach, B., O'Connell, S., Bock, K.W., Minai, M., et al. (2020). Evaluation of the mRNA-1273 Vaccine against SARS-CoV-2 in Nonhuman Primates. N. Engl. J. Med. *383*, 1544–1555. https://doi.org/10.1056/NEJMoa2024671.
29. Chandrashekar, A., Liu, J., Yu, J., McMahan, K., Tostanoski, L.H., Jacob-Dolan, C., Mercado, N.B., Anioke, T., Chang, A., Gardner, S., et al. (2021). Prior infection with SARS-CoV-2 WA1/2020 partially protects rhesus macaques against reinfection with B.1.1.7 and B.1.351 variants. Sci. Transl. Med. *13*, eabj2641. https://doi.org/10.1126/scitranslmed.abj2641.
30. Jacob-Dolan, C., Yu, J., McMahan, K., Giffin, V., Chandrashekar, A., Martinot, A.J., Anioke, T., Powers, O.C., Hall, K., Hope, D., et al. (2023). Immunogenicity and protective efficacy of GBP510/AS03 vaccine against SARS-CoV-2 delta challenge in rhesus macaques. NPJ Vaccines *8*, 23. https://doi.org/10.1038/s41541-023-00622-0.
31. Chandrashekar, A., Yu, J., McMahan, K., Jacob-Dolan, C., Liu, J., He, X., Hope, D., Anioke, T., Barrett, J., Chung, B., et al. (2022). Vaccine protection against the SARS-CoV-2 Omicron variant in macaques. Cell *185*, 1549–1555.e11. https://doi.org/10.1016/j.cell.2022.03.024.
32. Yu, J., Thomas, P.V., Sciacca, M., Wu, C., Liu, J., He, X., Miller, J., Hachmann, N.P., Surve, N., McMahan, K., et al. (2023). Ad26.COV2.S and SARS-CoV-2 spike protein ferritin nanoparticle vaccine protect against SARS-CoV-2 Omicron BA.5 challenge in macaques. Cell Rep. Med. *4*, 101018. https://doi.org/10.1016/j.xcrm.2023.101018.
33. Khare, S., Gurry, C., Freitas, L., Schultz, M.B., Bach, G., Diallo, A., Akite, N., Ho, J., Lee, R.T., Yeo, W., et al. (2021). GISAID's Role in Pandemic Response. China CDC Wkly. *3*, 1049–1051. https://doi.org/10.46234/ccdcw2021.255.

CellPress
OPEN ACCESS

34. Aksamentov, I., Roemer, C., Hodcroft, E., and Neher, R. (2021). Nextclade: clade assignment, mutation calling and quality control for viral genomes. J. Open Source Softw. *6*, 3773. https://doi.org/10.21105/joss.03773.
35. Bloom, J.D. (2024). https://github.com/jbloom/SARS2-clade-spike-diffs.
36. Wilks, S.H., Mühlemann, B., Shen, X., Türeli, S., LeGresley, E.B., Netzl, A., Caniza, M.A., Chacaltana-Huarcaya, J.N., Corman, V.M., Daniell, X., et al. (2023). Mapping SARS-CoV-2 antigenic relationships and serological responses. Science *382*, eadj0070. https://doi.org/10.1126/science.adj0070.
37. Mykytyn, A.Z., Rosu, M.E., Kok, A., Rissmann, M., van Amerongen, G., Geurtsvankessel, C., de Vries, R.D., Munnink, B.B.O., Smith, D.J., Koopmans, M.P.G., et al. (2023). Antigenic mapping of emerging SARS-CoV-2 omicron variants BM.1.1.1, BQ.1.1, and XBB.1. Lancet Microbe *4*, e294–e295. https://doi.org/10.1016/s2666-5247(22)00384-6.
38. Rössler, A., Netzl, A., Knabl, L., Schäfer, H., Wilks, S.H., Bante, D., Falkensammer, B., Borena, W., von Laer, D., Smith, D.J., and Kimpel, J. (2022). BA.2 and BA.5 omicron differ immunologically from both BA.1 omicron and pre-omicron variants. Nat. Commun. *13*, 7701. https://doi.org/10.1038/s41467-022-35312-3.
39. Rössler, A., Knabl, L., von Laer, D., and Kimpel, J. (2022). Neutralization Profile after Recovery from SARS-CoV-2 Omicron Infection. N. Engl. J. Med. *386*, 1764–1766. https://doi.org/10.1056/NEJMc2201607.
40. Evans, J.P., Zeng, C., Qu, P., Faraone, J., Zheng, Y.M., Carlin, C., Bednash, J.S., Zhou, T., Lozanski, G., Mallampalli, R., et al. (2022). Neutralization of SARS-CoV-2 Omicron sub-lineages BA.1, BA.1.1, and BA.2. Cell Host Microbe *30*, 1093–1102.e3. https://doi.org/10.1016/j.chom.2022.04.014.
41. Tortorici, M.A., Addetia, A., Seo, A.J., Brown, J., Sprouse, K., Logue, J., Clark, E., Franko, N., Chu, H., and Veesler, D. (2024). Persistent immune imprinting occurs after vaccination with the COVID-19 XBB.1.5 mRNA booster in humans. Immunity *57*, 904–911.e4. https://doi.org/10.1016/j.immuni.2024.02.016.
42. Jeworowski, L.M., Mühlemann, B., Walper, F., Schmidt, M.L., Jansen, J., Krumbholz, A., Simon-Lorière, E., Jones, T.C., Corman, V.M., and Drosten, C. (2024). Humoral immune escape by current SARS-CoV-2 variants BA.2.86 and JN.1, December 2023. Euro Surveill. *29*, 2300740. https://doi.org/10.2807/1560-7917.Es.2024.29.2.2300740.
43. Kaku, Y., Yo, M.S., Tolentino, J.E., Uriu, K., Okumura, K., Ito, J., and Sato, K.; Genotype to Phenotype Japan G2P-Japan Consortium (2024). Virological characteristics of the SARS-CoV-2 KP.3, LB.1, and KP.2.3 variants. Lancet Infect. Dis. *24*, e482–e483. https://doi.org/10.1016/s1473-3099(24)00415-8.
44. Kaku, Y., Uriu, K., Kosugi, Y., Okumura, K., Yamasoba, D., Uwamino, Y., Kuramochi, J., Sadamasu, K., Yoshimura, K., Asakura, H., et al. (2024). Virological characteristics of the SARS-CoV-2 KP.2 variant. Lancet Infect. Dis. *24*, e416. https://doi.org/10.1016/s1473-3099(24)00298-6.
45. Li, P., Faraone, J.N., Hsu, C.C., Chamblee, M., Zheng, Y.M., Carlin, C., Bednash, J.S., Horowitz, J.C., Mallampalli, R.K., Saif, L.J., et al. (2024). Neutralization escape, infectivity, and membrane fusion of JN.1-derived SARS-CoV-2 SLip, FLiRT, and KP.2 variants. Cell Rep. *43*, 114520. https://doi.org/10.1016/j.celrep.2024.114520.
46. Rössler, A., Netzl, A., Knabl, L., Bante, D., Wilks, S.H., Borena, W., von Laer, D., Smith, D.J., and Kimpel, J. (2023). Characterizing SARS-CoV-2 neutralization profiles after bivalent boosting using antigenic cartography. Nat. Commun. *14*, 5224. https://doi.org/10.1038/s41467-023-41049-4.
47. van der Straten, K., Guerra, D., van Gils, M.J., Bontjer, I., Caniels, T.G., van Willigen, H.D.G., Wynberg, E., Poniman, M., Burger, J.A., Bouhuijs, J.H., et al. (2022). Antigenic cartography using sera from sequence-confirmed SARS-CoV-2 variants of concern infections reveals antigenic divergence of Omicron. Immunity *55*, 1725–1731.e4. https://doi.org/10.1016/j.immuni.2022.07.018.
48. ECDC. Community Network of Reference Laboratories (CNRL) for Human Influenza in Europe. SURVEILLANCE REPORT. Influenza virus characterisation. Summary Europe, February 2011. (2011). https://www.ecdc.europa.eu/sites/default/files/media/en/publications/Publications/1103_Influenza_virus_characterisation_2011_March.pdf.
49. Cao, Y., Wang, J., Jian, F., Xiao, T., Song, W., Yisimayi, A., Huang, W., Li, Q., Wang, P., An, R., et al. (2022). Omicron escapes the majority of existing SARS-CoV-2 neutralizing antibodies. Nature *602*, 657–663. https://doi.org/10.1038/s41586-021-04385-3.
50. Cao, Y., Su, B., Guo, X., Sun, W., Deng, Y., Bao, L., Zhu, Q., Zhang, X., Zheng, Y., Geng, C., et al. (2020). Potent Neutralizing Antibodies against SARS-CoV-2 Identified by High-Throughput Single-Cell Sequencing of Convalescent Patients' B Cells. Cell *182*, 73–84.e16. https://doi.org/10.1016/j.cell.2020.05.025.
51. Kim, S.I., Noh, J., Kim, S., Choi, Y., Yoo, D.K., Lee, Y., Lee, H., Jung, J., Kang, C.K., Song, K.-H., et al. (2021). Stereotypic neutralizing V_H antibodies against SARS-CoV-2 spike protein receptor binding domain in patients with COVID-19 and healthy individuals. Sci. Transl. Med. *13*, eabd6990. https://doi.org/10.1126/scitranslmed.abd6990.
52. He, W.-t., Yuan, M., Callaghan, S., Musharrafieh, R., Song, G., Silva, M., Beutler, N., Lee, W.-H., Yong, P., Torres, J.L., et al. (2022). Broadly neutralizing antibodies to SARS-related viruses can be readily induced in rhesus macaques. Sci. Transl. Med. *14*, eabl9605. https://doi.org/10.1126/scitranslmed.abl9605.
53. Feng, Y., Yuan, M., Powers, J.M., Hu, M., Munt, J.E., Arunachalam, P.S., Leist, S.R., Bellusci, L., Kim, J., Sprouse, K.R., et al. (2023). Broadly neutralizing antibodies against sarbecoviruses generated by immunization of macaques with an AS03-adjuvanted COVID-19 vaccine. Sci. Transl. Med. *15*, eadg7404. https://doi.org/10.1126/scitranslmed.adg7404.
54. Yang, S., Yu, Y., Jian, F., Song, W., Yisimayi, A., Chen, X., Xu, Y., Wang, P., Wang, J., Yu, L., et al. (2023). Antigenicity and infectivity characterisation of SARS-CoV-2 BA.2.86. Lancet Infect. Dis. *23*, e457–e459. https://doi.org/10.1016/s1473-3099(23)00573-x.
55. Planas, D., Staropoli, I., Michel, V., Lemoine, F., Donati, F., Prot, M., Porrot, F., Guivel-Benhassine, F., Jeyarajah, B., Brisebarre, A., et al. (2024). Distinct evolution of SARS-CoV-2 Omicron XBB and BA.2.86/JN.1 lineages combining increased fitness and antibody evasion. Nat. Commun. *15*, 2254. https://doi.org/10.1038/s41467-024-46490-7.
56. Liang, C.-Y., Raju, S., Liu, Z., Li, Y., Asthagiri Arunkumar, G., Case, J.B., Scheaffer, S.M., Zost, S.J., Acreman, C.M., Gagne, M., et al. (2024). Imprinting of serum neutralizing antibodies by Wuhan-1 mRNA vaccines. Nature *630*, 950–960. https://doi.org/10.1038/s41586-024-07539-1.
57. Patel, N., Trost, J.F., Guebre-Xabier, M., Zhou, H., Norton, J., Jiang, D., Cai, Z., Zhu, M., Marchese, A.M., Greene, A.M., et al. (2023). XBB.1.5 spike protein COVID-19 vaccine induces broadly neutralizing and cellular immune responses against EG.5.1 and emerging XBB variants. Sci. Rep. *13*, 19176. https://doi.org/10.1038/s41598-023-46025-y.
58. FDA (2024). Updated COVID-19 Vaccines for Use in the United States Beginning in Fall 2024. https://www.fda.gov/vaccines-blood-biologics/updated-covid-19-vaccines-use-united-states-beginning-fall-2024.
59. Happle, C., Hoffmann, M., Kempf, A., Nehlmeier, I., Stankov, M.V., Calderon Hampel, N., Witte, T., Pöhlmann, S., Behrens, G.M.N., and Dopfer-Jablonka, A. (2024). Humoral immunity after mRNA SARS-CoV-2 omicron JN.1 vaccination. Lancet Infect. Dis. *24*, e674–e676. https://doi.org/10.1016/s1473-3099(24)00603-0.
60. Wilks, S. (2023). acorg/mapping_SARS-CoV-2_antigenic_relationships_and_serological_responses: Mapping SARS-CoV-2 Antigenic Relationships and Serological Responses, First Release (v.1.0.0) (Zenodo). https://doi.org/10.5281/zenodo.8195839.
61. Netzl, A. (2023). acorg/roessler_netzl_et_al2023: Manuscript accepted (v1.0.0) (Zenodo). https://doi.org/10.5281/zenodo.8199367.
62. Strizki, J.M., Gaspar, J.M., Howe, J.A., Hutchins, B., Mohri, H., Nair, M.S., Kinek, K.C., McKenna, P., Goh, S.L., and Murgolo, N. (2024). Molnupiravir maintains antiviral activity against SARS-CoV-2 variants and exhibits a high barrier to the development of resistance. Antimicrob. Agents Chemother. *68*, e0095323. https://doi.org/10.1128/aac.00953-23.

63. Wilks S.H. Racmacs: R Antigenic Cartography Macros 2022. https://acorg.github.io/Racmacs, https://github.com/acorg/Racmacs.

64. Wilks, S.H. (2021). ablandscapes: Making Antibodz landscapes Using R. https://github.com/acorg/ablandscapes.

65. Team, C. (2021). R: A Language and Environment for Statistical Computing. https://www.R-project.org.

66. Gabry, J., and Češnovar, R. (2022). R Interface to ‘CmdStan. https://mc-stan.org/cmdstanr/.

67. Kassambara, A. rstatix: Pipe-Friendly Framework for Basic Statistical Tests. https://CRAN.R-project.org/package=rstatix.

68. Wickham, H. (2016). ggplot2: Elegant Graphics for Data Analysis, 2 Edition (Springer Cham). https://doi.org/10.1007/978-3-319-24277-4.

69. Wickham, H., Francois, R., Henry, L., Mueller, K., and Vaughan, D. dplyr: A Grammar of Data Manipulation_. R package version 1.1.0. https://CRAN.R-project.org/package=dplyr.

70. Wickham, H., Averick, M., Bryan, J., Chang, W., McGowan, L., François, R., Grolemund, G., Hayes, A., Henry, L., Hester, J., et al. (2019). Welcome to the tidyverse. J. Open Source Softw. *4*, 1686. https://doi.org/10.21105/joss.01686.

71. Pedersen, T.L. (2024). patchwork: The Composer of Plots. R package version 1.3.0. https://CRAN.R-project.org/package=patchwork.

72. Kassambara, A. (2023). ggpubr: “ggplot2” Based Publication Ready Plots_. R package version 0.6.0. https://CRAN.R-project.org/package=ggpubr.

73. Netzl, A. (2024). GitHub repository.acorg/roessler_netzl_et_al2024: Manuscript accepted (v1.0) (Zenodo). https://github.com/acorg/roessler_netzl_et_al2024.git.

74. Wilks, S.H. titertools: A statistical toolkit for the analysis of censored titration data. https://github.com/shwilks/titertools.git.

STAR★METHODS

KEY RESOURCES TABLE

REAGENT or RESOURCE	SOURCE	IDENTIFIER
Bacterial and virus strains		
SARS-CoV-2 (Wuhan)	BEI resources	Isolate: hCoV-19/USA-WA1/2020
SARS-CoV-2 (Alpha; B.1.1.7)	BEI resources	Isolate: hCov-19/USA/CA_CDC_5574/2020
SARS-CoV-2 (Beta; B.1.351)	BEI resources	Isolate: hCov-19/South Africa/KRISP-K005325/2020
SARS-CoV-2 (Gamma; P.1)	BEI resources	Isolate: hCoV-19/Japan/IC-0564/2021
SARS-CoV-2 (Delta; B.1.617.2)	BEI resources	Isolate: hCoV-19/USA/MD-HP05647/2021
SARS-CoV-2 (BA.1; B.1.1.529)	BEI resources	Isolate: hCoV-19/USA/MD-HP20874/2021
SARS-CoV-2 (BA.2.12.1)	BEI resources	Isolate: hCoV-19/USA/NY-MSHSPSP-PV56475/2022
SARS-CoV-2 (BA.4)	Strizki et al.[62]	Isolate: hCoV-19/USA/MDHP30386/2022
SARS-CoV-2 (BA.5)	Strizki et al.[62]	Isolate: hCov-19/USA/COR-22-063113/2022
Biological samples		
Non-human primate serum samples	Bioqual, Inc.	N/A
Critical commercial assays		
Steady-Glo® Luciferase Assay System	Promega	Cat# 2550
Deposited data		
Neutralization data of Human dataset 1	Rössler et al.[46,61]	Zenodo: https://doi.org/10.5281/zenodo.8199367
Neutralization data of Human dataset 2	Wilks et al.[36,60]	Zenodo: https://doi.org/10.5281/zenodo.8195839
Neutralization data of NHP samples	This study	https://github.com/acorg/roessler_netzl_et_al2024.git, Zenodo: https://doi.org/10.5281/zenodo.14217590
Experimental models: Cell lines		
HEK293T	ATCC	CRL_3216; RRID:CVCL_0063
HEK293T-ACE2	In house[26]	N/A
Experimental models: Organisms/strains		
Rhesus or cynomolgus macaques	Bioqual, Inc.	N/A
Recombinant DNA		
psPAX2	Addgene	Cat# 12260; RRID:Addgene_12260
Plasmid: pLenti-CMVPuro-Luc	Addgene	Cat# 17477: RRID:Addgene_17477
pcDNA3.1(+) Wuhan (WA1/2020) spike ΔCT	GISAID ID: EPI_ISL_402124	N/A
pcDNA3.1(+) B.1.1.7 spike ΔCT	GISAID ID: EPI_ISL_601443	N/A
pcDNA3.1(+) B.1.351 spike ΔCT	GISAID ID: EPI_ISL_712096	N/A
pcDNA3.1(+) B.1.617.2 spike ΔCT	GISAID ID: EPI_ISL_2020950	N/A
pcDNA3.1(+) BA.1 spike ΔCTeΔ	GISAID ID: EPI_ISL_7358094.2	N/A
pcDNA3.1(+) BA.2 spike ΔCT	GISAID ID: EPI_ISL_6795834.2	N/A
pcDNA3.1(+) BA.2.12.1 spike ΔCT	GISAID ID: EPI_ISL_12003853.1	N/A
pcDNA3.1(+) CH.1.1 spike ΔCT	GISAID ID: EPI_ISL_17202004	N/A
pcDNA3.1(+) DV.7.1 spike ΔCT	GISAID ID: EPI_ISL_18052118	N/A
pcDNA3.1(+) BA.5 spike ΔCT	GISAID ID: EPI_ISL_12268495.2	N/A
pcDNA3.1(+) BQ.1.1 spike ΔCT	GISAID ID: EPI_ISL_14752457	N/A
pcDNA3.1(+) XBB.1 spike ΔCT	GISAID ID: EPI_ISL_15232105	N/A
pcDNA3.1(+) XBB.1.5 spike ΔCT	GISAID ID: EPI_ISL_16418320	N/A
pcDNA3.1(+) XBC.1.6 spike ΔCT	GISAID ID: EPI_ISL_17851490	N/A

(*Continued on next page*)

Continued		
REAGENT or RESOURCE	SOURCE	IDENTIFIER
pcDNA3.1(+) FL.1.5.1 spike ΔCT	GISAID ID: EPI_ISL_18126515	N/A
pcDNA3.1(+) HV.1 spike ΔCT	GISAID ID: EPI_ISL_18592608	N/A
pcDNA3.1(+) HK.3 spike ΔCT	GISAID ID: EPI_ISL_18631954	N/A
pcDNA3.1(+) EG.5.1 spike ΔCT	GISAID ID: EPI_ISL_17976635	N/A
pcDNA3.1(+) BA.2.86 spike ΔCT	GISAID ID: EPI_ISL_18121060	N/A
pcDNA3.1(+) JN.1 spike ΔCT	GISAID ID: EPI_ISL_18680594	N/A
pcDNA3.1(+) KP.2 spike ΔCT	GISAID ID: EPI_ISL_19189039	N/A
pcDNA3.1(+) KP.3 spike ΔCT	GISAID ID: EPI_ISL_19203968	N/A
pcDNA3.1(+) KZ.1.1.1 spike ΔCT	GISAID ID: EPI_ISL_19028189	N/A
Software and algorithms		
Illustrator	Adobe	RRID:SCR_010279
Racmacs package for antigenic cartography	Wilks et al.[63]	https://acorg.github.io/Racmacs, https://github.com/acorg/Racmacs
Antibody landscapes	Wilks et al.[64]	https://github.com/acorg/ablandscapes
Bayesian modeling framework	Mühlemann et al.[19]	https://doi.org/10.5281/zenodo.10948979
R: A Language and Environment for Statistical Computing	R Core Team[65]	https://www.R-project.org
Cmdstanr	Gabry and Češnovar[66]	https://mc-stan.org/cmdstanr/, https://discourse.mc-stan.org
rstatix	Kassambara[67]	https://CRAN.R-project.org/package=rstatix
ggplot2 v 3.5.1	Wickham[68]	https://ggplot2.tidyverse.org
dplyr v 1.1.0	Wickham[69]	https://CRAN.R-project.org/package=dplyr
tidyverse v 2.0.0	Wickham et al.[70]	https://doi.org/10.21105/joss.01686
patchwork v 1.3.0	Pedersen[71]	https://CRAN.R-project.org/package=patchwork
ggpubr v 0.6.0	Kassambra[72]	https://CRAN.R-project.org/package=ggpubr

EXPERIMENTAL MODEL AND STUDY PARTICIPANT DETAILS

Animals

In this study, 113 serum samples from 91 outbred adult male and female rhesus or cynomolgus macaques (6–10 years old) housed at BIOQUAL Inc., were analyzed. Animals were randomly allocated and the studies were conducted in compliance with all relevant local, state, and federal regulations and were approved by the BIOQUAL Institutional Animal Care and Use Committee. Samples were mainly collected as part of previous studies[26,29–32] and sera were grouped according to SARS-CoV-2 exposure histories. The study cohorts comprised 71 previously naive animals, that have been infected with a SARS-CoV-2 variant (Wuhan, Alpha, Beta, Gamma, Delta, Omicron BA.1, BA.2.12.1 or BA.4/BA.5) or immunized with monovalent Wuhan, Beta or XBB.1.5 adenovirus vector or mRNA vaccines (n = 20). Virus challenge was routinely performed intranasally and intratracheally at a dose range of $1x10^4$ to $1x10^6$ plaque-forming units (PFU) and sera were collected 10 to 28 days later. The samples from vaccinated animals were taken 26 days after animals received a single dose (5×10^{10} viral particles) of a Wuhan (Ad.26.COV.S, Janssen) or Beta variant (Ad.26.COV.S.351, Janssen) adenoviral vector vaccine, or 14 days after two homologous immunizations (each 30 μg) with a monovalent Wuhan or XBB.1.5 adapted mRNA vaccine (BNTb162b, Pfizer). A detailed overview of the study cohorts' characteristics is given in Tables S2 and S3.

Cell lines

All cells were cultured at 37°C in a humified atmosphere with 5% CO_2. HEK293T cells were cultured in modified Eagle's medium (DMEM, Gibco) supplemented with 10% fetal bovine serum (FBS, Omega Scientific, Inc.), 1% penicillin-streptomycin (Gibco). HEK293T-ACE2 cells were additionally selected with 1 μg/mL puromycin (Sigma-Aldrich).

METHOD DETAILS

Pseudovirus neutralization assay

Heat-inactivated sera were tested for neutralization against a panel of 23 SARS-CoV-2 variants as previously described employing a lentivirus-based pseudo-typed virus encoding for a luciferase reporter gene. Analyzed SARS-CoV-2 variants comprise pre-Omicron

(Wuhan, Alpha, Beta, Delta), early Omicron (BA.1, BA.2, BA.2.12.1, CH.1.1, DV.7.1, BA.5, BQ.1.1), XBC.1.6 and XBB.1-descended variants (XBB.1, XBB.1.5, FL.1.5.1, HV.1, HK.3, EG.5), and recent saltation Omicron variants (BA.2.86, JN.1, KP.2, KP.3 and KZ.1.1.1) (Table S1). A phylogenetic tree of analyzed virus variants as well as spike mutation profiles are shown in Figure 1. Briefly, human embryonic kidney HEK293T cells (ATCC CRL_3216) were co-transfected with a luciferase reporter plasmid (pLenti-CMV Puro-Luc, Addgene), packaging construct psPAX2 (AIDS Resource and Reagent Program) and Spike protein expressing pcDNA3.1-SARS-CoV-2 SΔCT using lipofectamine 2000 (ThermoFisher Scientific). The spike sequences for analyzed SARS-CoV-2 variants were retrieved from GISAID and corresponding IDs are listed in Table S1. The pseudovirus containing supernatants were collected two days after transfection and filtered through a 0.45-μm Filter. To determine neutralization titers, 3-fold dilutions of the sera were pre-incubated with the same volume of pseudovirus for 1h at 37°C before the sera/pseudovirus-mix was transferred to HEK293T-ACE2 cells seeded the day before (2×10^4 cells/well). Approximately 48h later, cells were lysed using Steady-Glo Luciferase System (Promega) according to manufacturer's instructions. The continuous 50% neutralization titers (IC_{50}) were calculated by non-linear regression using GraphPad Prism software 9.5.1. NAb titers $\leq$1:1 were set to 1:1, and titers >1:20 were considered positive.

Antigenic cartography

Antigenic cartography is frequently used to describe antigenic relationships of virus strains and visualize antibody profiles as antibody landscapes.[9,19,36,37,46,54] To construct an antigenic map, fold changes of neutralization titers from the maximum titer variant are calculated for each serum-variant pair, corresponding to the so-called table distance. Then, serum-variant Euclidean map distances are optimized such that map distance reflects the table distance and map stress, the error between table and map distance, is minimized. One unit map distance corresponds to one 2-fold dilution of neutralization titers. Detailed algorithm descriptions are given by Smith et al.[9] and the Racmacs reference page.[63] Maps were constructed in R[65] (v 4.2.2) with the Racmacs package[63] (v 1.1.35), with 1000 optimizations and the options "list(ignore_disconnected = TRUE, dim_annealing = TRUE)" as variation was found in some points when setting "dim_annealing = FALSE". Titers below the limit of detection (LOD) 20 were set to "<20" in the titer table as Racmacs handles uncertainty due to <LOD titers. The number of sera and serum groups used to construct the map are given in the legend in Figure 3. Map diagnostics were performed as previously described[38,46] and results are stored in the GitHub repository.[73] Antibody landscapes were constructed with the ablandscapes package[64] (v 1.1.0). To construct an antibody landscape, the x- and y-coordinates and slope of a single-cone surface are fitted to neutralization titers, where titers against a specific variant are plotted in a third dimension on top of the variant in the x-y-plane given by an antigenic map. The slope was fit per serum group using the "ablandscape.fit" function with parameters "bandwidth = 1, degree = 1, method = "cone", error.sd = 1, control = list(optimise.cone.slope = TRUE)". Geometric mean titers (GMTs) were calculated using the titertools R package[74] (v 0.0.0.9001).

Reactivity adjustments

Alpha titers were higher than homologous titers indicating a high reactivity variant. To quantify and control for that, the Racmacs function[63] "optimizeAgReactivity" with the same options as for map optimization was used. This function optimizes antigen reactivity to minimize map stress, i.e., the error between measured titers and mapped serum and variant coordinates. Alpha titers were optimized to a reactivity adjustment of −0.84 on the log2 scale (all Alpha titers were multiplied by 0.56 for map making). This resulted in a map stress reduction from 919.78 in the unadjusted map to 892.77 in the adjusted map.

Titer modeling

To compare titers from NHP and humans we employed a Bayesian modeling framework that adjusts for titer magnitude differences based on individual serum, organism and assay reactivity. The approach was developed by Mühlemann et al.[19] to evaluate SARS-CoV-2 titer patterns from different laboratories and organisms and recently employed to compare hamster and human titrations from the same laboratory.[20] Here, we adapted their framework to control for and quantify titer differences based on organism (Human vs. NHP) and assay (authentic virus in Vero-TMPRSS2/ACE2 vs. lentivirus pseudotype in 293T/ACE2).

Each titer is modeled as a combination of geometric mean titer (GMT) per variant and serum group, a reactivity effect of each individual serum, an organism-specific reactivity effect and an assay-dependent reactivity effect, where reactivity describes a factor by which titers against all variants are multiplied.

$$logtiter_{ijma} = serumGroupGMT_{iJ} + serumEffect_j + organismEffect_m + assayEffect_a + \varepsilon_{ij} \quad \text{Equation (1)}$$

In Equation 1, the *serumGroupGMT* refers to the log2 titer of antigen *i* in serum group *J,* the *serumEffect* corresponds to the reactivity bias of serum *j,* the *organismEffect* to the reactivity bias of organism *m*, the *assayEffect* the reactivity bias of assay *a*, and ε to log2 normally distributed noise for each measurement, where the standard deviation σ is assumed to differ between datasets and is a parameter that is estimated.

The model was based on the cmdstanr model used by Mühlemann et al.[19] (R version 4.2.2,[65] cmdstanr version 0.5.3[66]), and priors for the standard deviation parameter σ were chosen based on their values (inverse gamma distribution, shape = 3, scale = 1.5). For modeling serum, organism and assay reactivity effects, the following distributions were used: serumGroupGMT: N(4, 20), serumEffect: N(0, 4), organismEffect: N(0, 4), assayEffect: N(0, 4). The mean of the posterior distributions was used to adjust the raw titers (Figure S8). cmdstanr's sampling, rather than its optimization function, was used as a comparison of values revealed a small difference between posterior means and optimized values (Figure S8) which can happen when the posterior distribution is not convex and

the optimization algorithm gets stuck in a local optimum.[66] The models were run for 1000 iterations with 1000 warmup iterations on 4 parallel chains and a maximum treedepth of 20. Cmdstanr's cmdstan_diagnose() function was used to evaluate model convergence, sampling size and independence and no problems were detected.

QUANTIFICATION AND STATISTICAL ANALYSIS

A Wilcoxon rank-sum test was performed to test for significant differences between Log2 neutralization titers of the two human datasets and the NHP dataset after adjusting for serum, assay and organism reactivity effects (Table S6). The non-parametric test was chosen as a Shapiro-Wilk test revealed that the distribution of Log2 titers differed significantly from a normal distribution. The tests were performed in R[65] (version 4.2.2) using the rstatix package[67] (version 0.7.2).

Cell Reports, Volume *44*

Supplemental information

Nonhuman primate antigenic cartography of SARS-CoV-2

Annika Rössler, Antonia Netzl, Ninaad Lasrado, Jayeshbhai Chaudhari, Barbara Mühlemann, Samuel H. Wilks, Janine Kimpel, Derek J. Smith, and Dan H. Barouch

Supplementary Figures

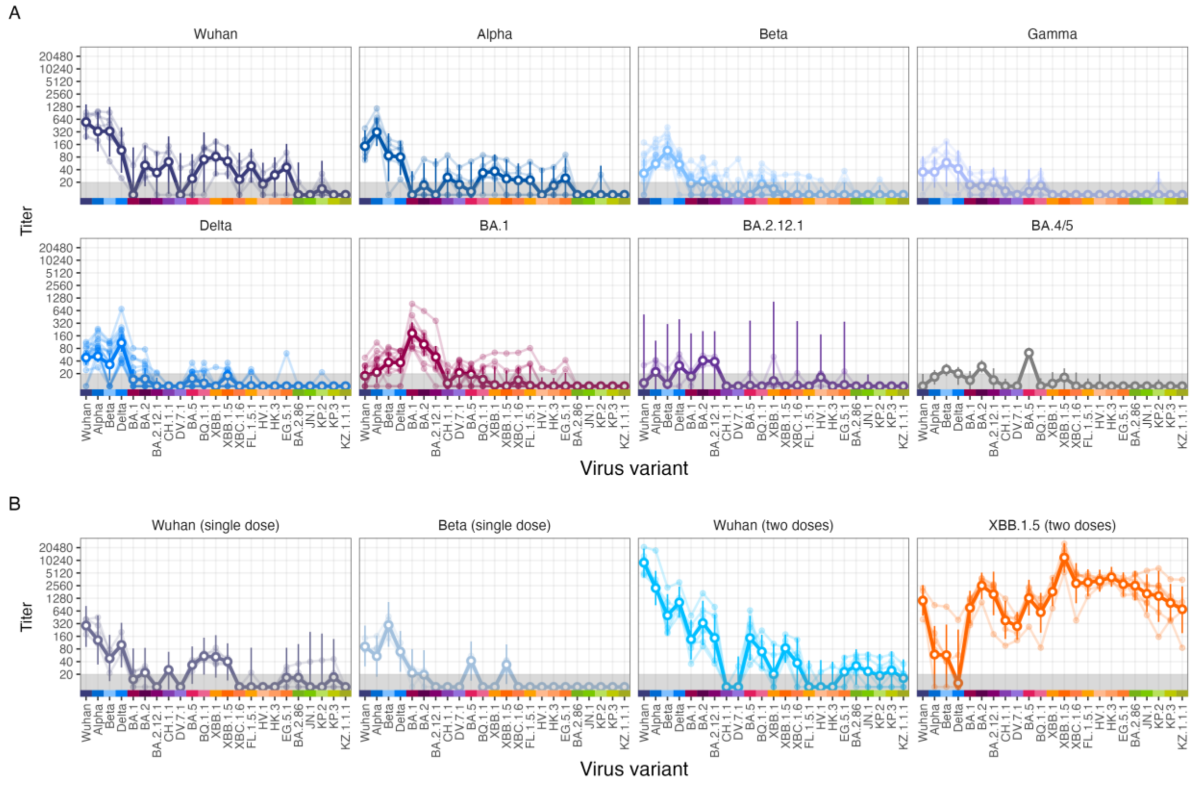

Figure S1. Titers with reactivity adjustment, related to Figure 2. A. Infected study cohorts by infecting virus variant and **B.** vaccine cohorts by vaccine regimen. Individual samples are shown as transparent lines with the geometric mean titer (GMT) given as solid line and the bars indicating 95% CI. The colors correspond to the colors in the map, the variant colors are given on the x axis. The numbers on top of each panel give the GMT against the respective variant in the top row and fold change from homologous in the bottom row. The GMT and fold changes were calculated using the titertools R package [1] (v.0.0.0.9001). Reactivity reduction of the Alpha variant was performed as described in the methods section; the raw Alpha titers were multiplied by a factor of 0.58.

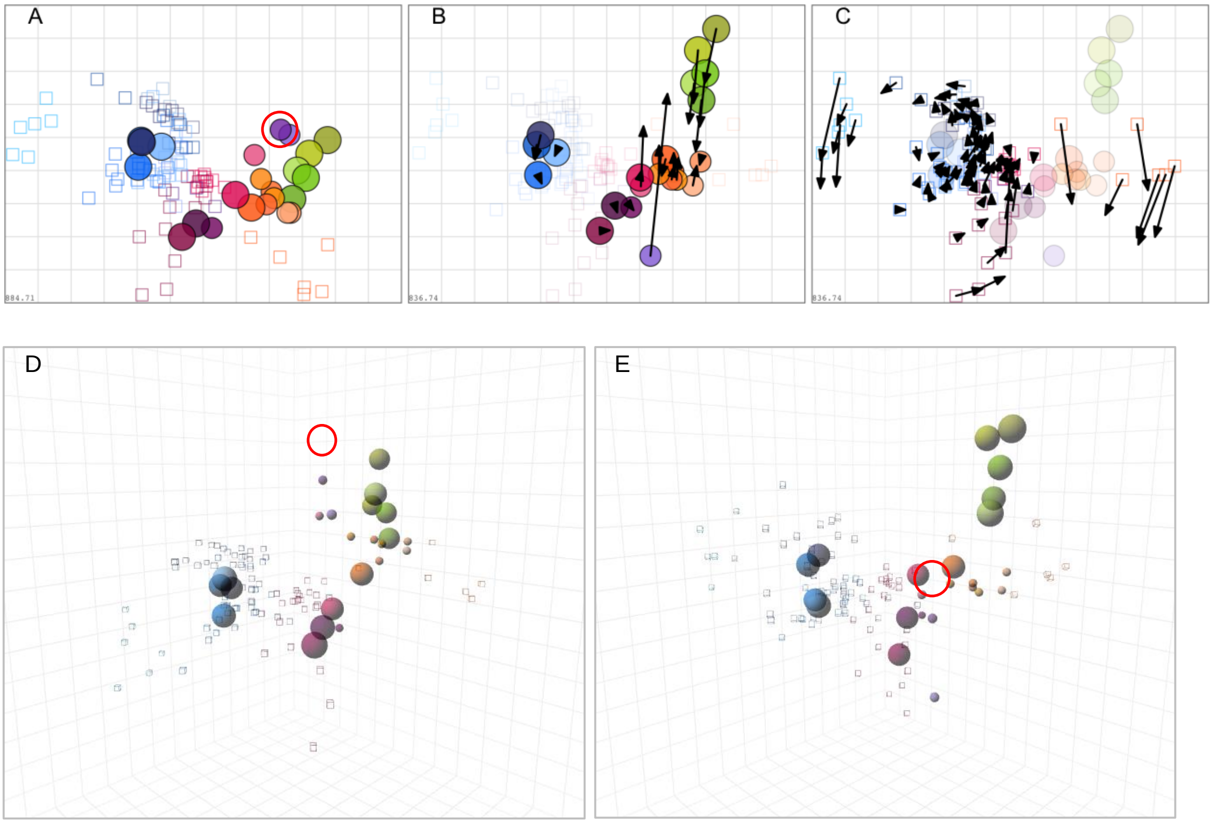

Figure S2. Bistable map including CH.1.1, related to Figure 3. A. A map including the highlighted CH.1.1 was constructed as described in the methods section, resulting in a different optimum. **B.** Arrows point from the variant's position in the map in fig.3A to the variant's position in the map in A. **C.** Arrows point from serum's position in the map in fig.3A to the variant's position in the map in A. **D, E.** The map in A was optimized in three dimensions and is shown in two different orientations. **D.** Shows a 3D orientation that would result in a map conformation as in **A** if projected onto 2D., **E.** shows a 3D orientation that would result in a map conformation as in **B** if projected onto 2D.

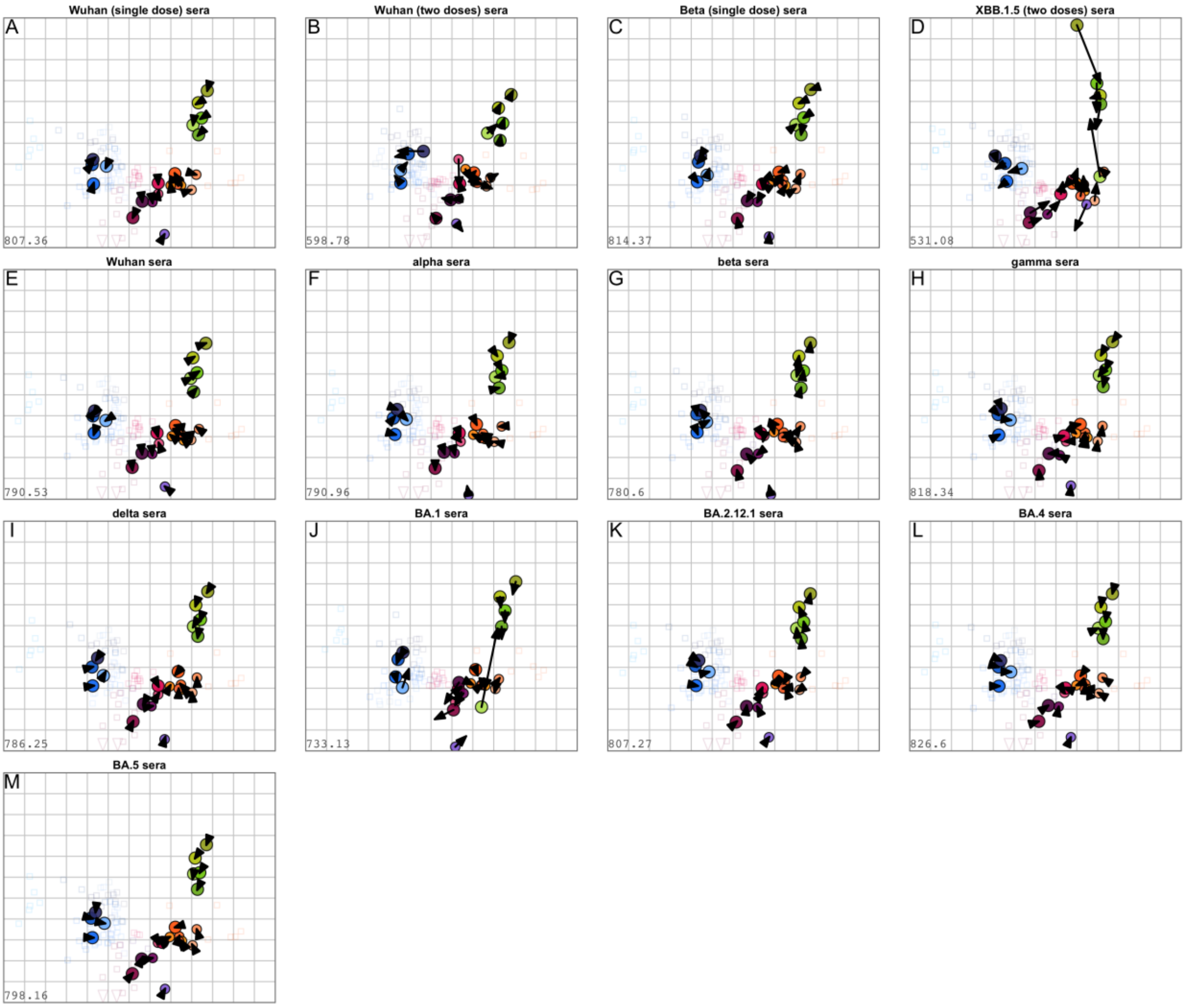

Figure S3. Assessing map robustness to the exclusion of sera, related to Figure 3. Each serum group was removed and the map reoptimized, with optimization settings as described in the Methods section. Arrows point to the position of each variant in the map shown in **figure 3**, for color correspondence refer to this map. A small arrow length indicates similar variant positions and map robustness to the exclusion of the particular serum group. Maps without sera vaccinated with **A.** Wuhan (single dose), **B.** Wuhan (two doses), **C.** Beta (single dose), **D.** XBB.1.5 (two doses), and infected with **E.** Wuhan, **F.** alpha, **G.** beta, **H.** gamma, **I.** delta, **J.** BA.1, **K.** BA.2.12.1, **L.** BA.4, or **M.** BA.5.

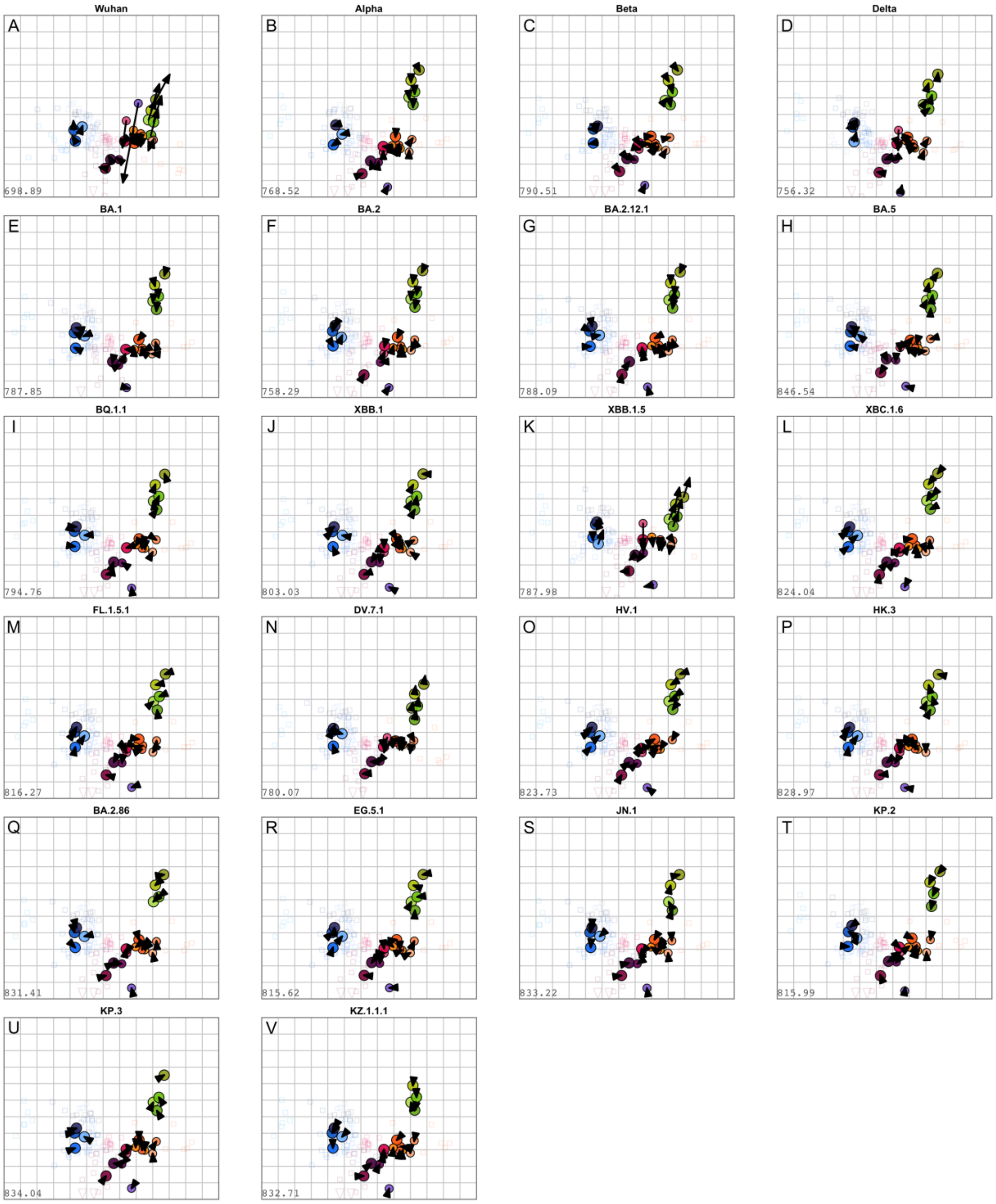

Figure S4. Assessing map robustness to the exclusion of antigen variants, related to Figure 3. Each antigen variant was removed and the map reoptimized, with optimization settings as described in the Methods section. Arrows point to the position of each variant in the map shown in **figure 3**, for color correspondence refer to this map. A small arrow length indicates similar variant positions and map robustness to the exclusion of the particular variant.

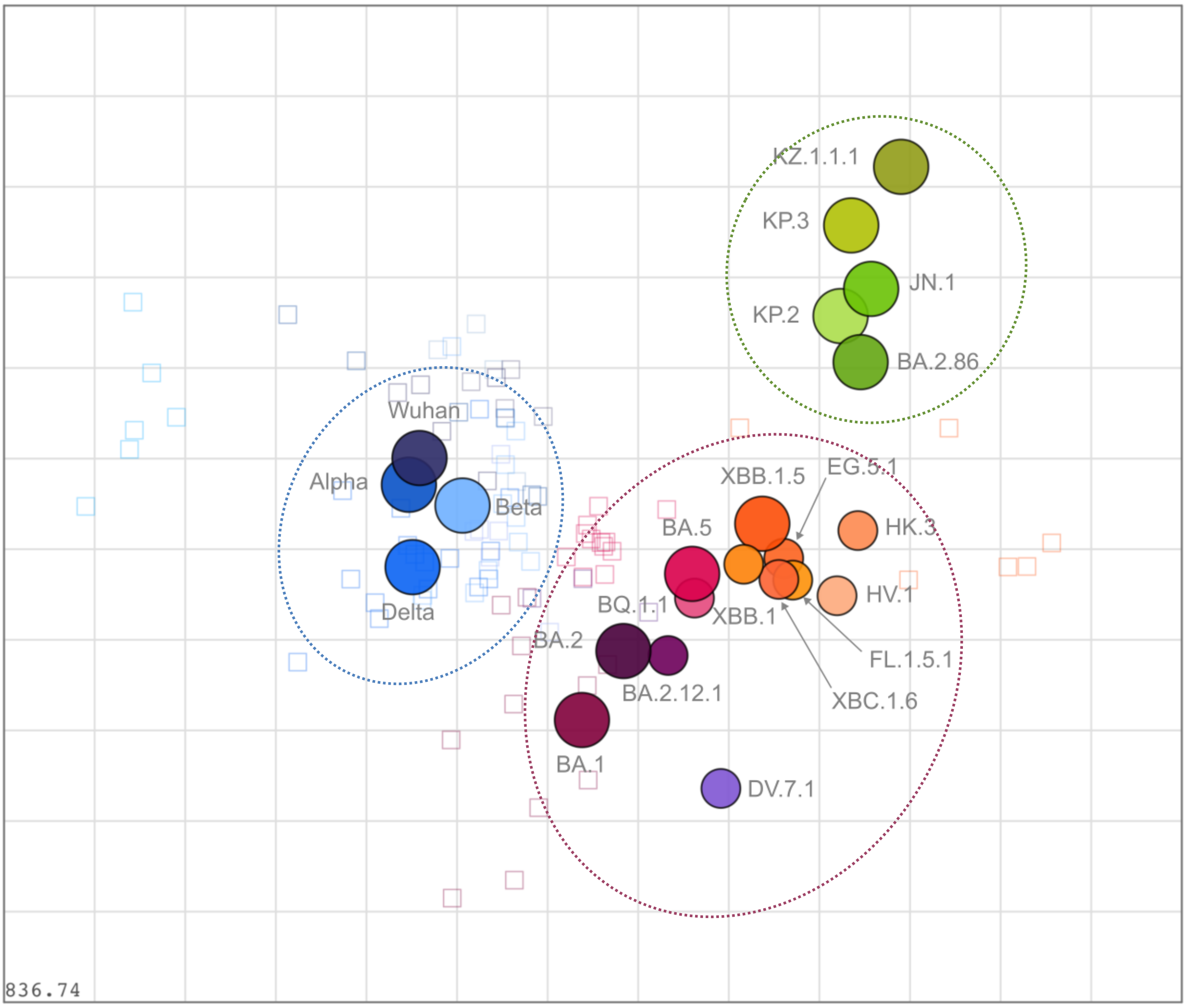

Figure S5. Zoomed-out version of the antigenic map, related to Figure 3. Virus variants are shown as colored circles, smaller circles denote sub-lineages, sera as open squares with the color corresponding to the infecting variant, vaccine sera are shown in grey tones. Dashed lines indicate three virus clusters; we grouped them according to their map position and clusters are highlighted by blue, purple/orange and green color schemes. The x- and y-axis represent antigenic distances with one grid square corresponding to one two-fold serum dilution of the neutralization titer. The map orientation within x- and y-axis is free as only relative distances can be inferred. Only single variant exposure sera and double vaccination sera have been used for construction of the map, the number of which is given in the legend of **figure 3**.

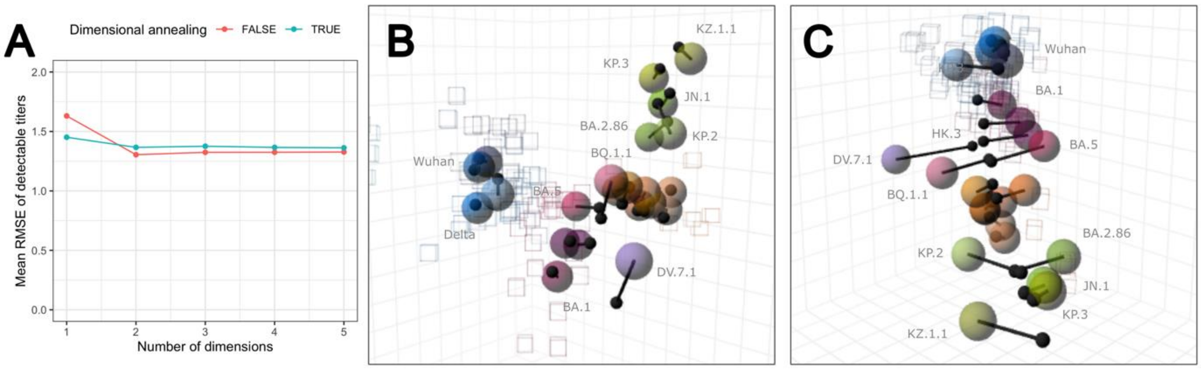

Figure S6. Map dimensionality, related to Figure 3. A Dimensionality test: Root Mean Squared Error (RMSE) between map and measured titers for detectable titers in 1 to 5 dimensions. Per dimension, 100 map replicates were constructed from 90% of measured titers with 1000 optimizations per replicate, once with the option "dim_annealing = TRUE" (blue) and once with "dim_annealing = FALSE" (red). The titers of the remaining 10% were predicted in each run and the RMSE calculated by comparing the predicted to the measured titers on the $\log_2$ scale. Both options were tested as the dimensional annealing with missing data did not find the global optimum, resulting in extremely similar RMSE across all dimensions [2]. **B, C** Front and side view of the map optimized in 3 dimensions with arrows pointing to the variants' position in the 2D map ("dim_annealing = TRUE").

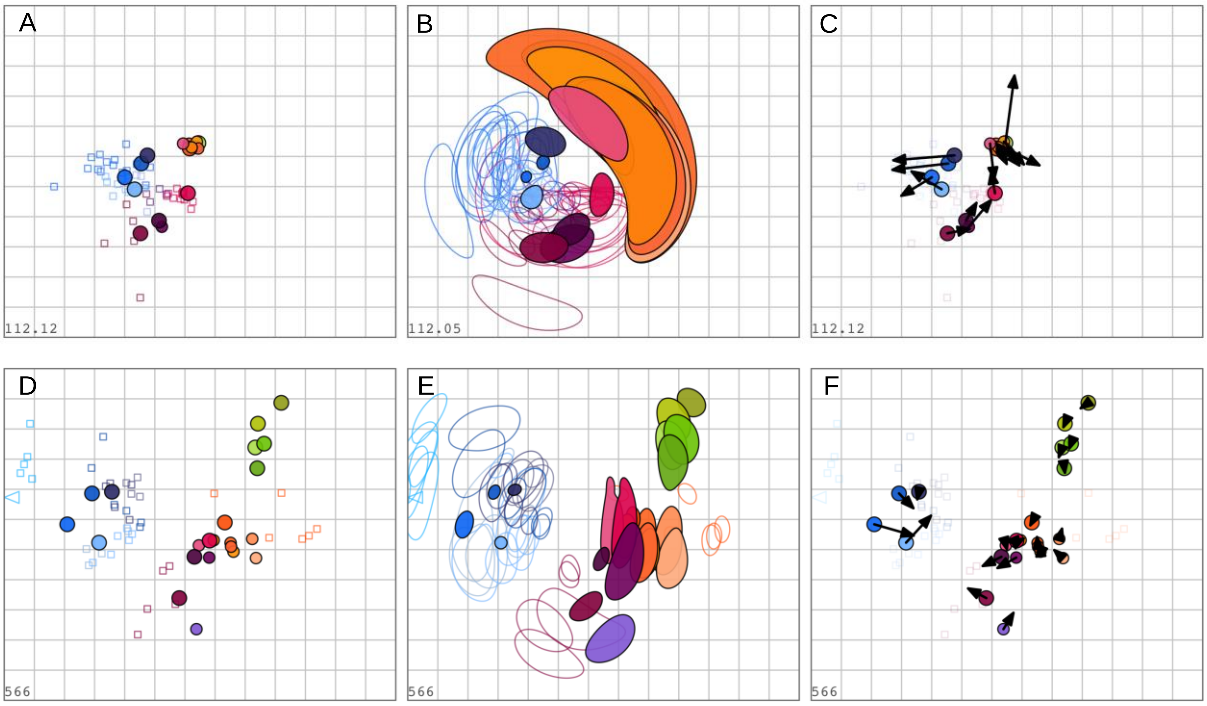

Figure S7. Antigenic maps from early and later sampled sera, related to Figure 3. Maps were constructed from sera sampled **A.** up to D10 post challenge and **D.** later than D10 post challenge. **B, E.** Constant force loci show the area which an item can occupy in the respective map without increasing map stress by more than one unit. **C, F.** Arrows point to the variants' positions in the main map. Descriptions apply to the map in the respective row.

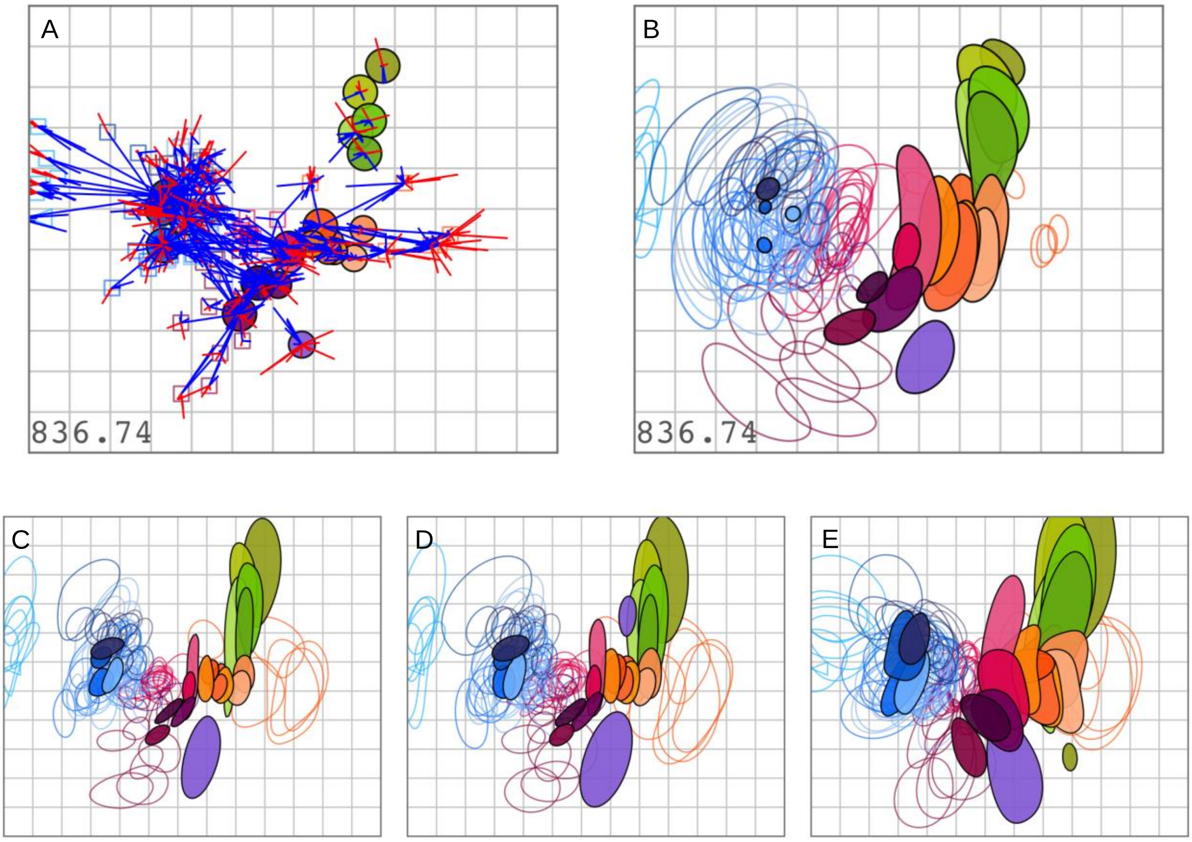

Figure S8. Map error and positional resolution, related to Figure 3. A. Error lines for each serum and antigen are shown in blue in case of larger map distance than target distance and red in case of smaller map distance than target distance. The length of each error bar indicates the magnitude of mismatch. Blue error lines point towards the variant-serum pair that has a smaller target distance; red error lines point away from the variant-serum pair. **B.** Constant force loci (Triangulation blobs) show the area for each serum and variant in which the item can move without increasing map stress by more than one unit. Filled shapes show variant Triangulation blobs, open shapes sera. **C-D.** 500 bootstrap repeats were performed with 1000 optimizations per repeat and options as listed in the Methods section. In each repeat, **C.** different weights are assigned to sera and antigen reactivity of the titer table. The weights are drawn randomly from a Dirichlet distribution. **D.** Sera and antigens are randomly resampled with replacement, and **E.** normally distributed noise with sd = 0.7 is added to antigens and individual titer measurements. The colored regions mark 68% (one standard deviation) of the positional variation for each variant (filled shapes) and sera (open shapes). The colors correspond to the colors used in **figure 3**.

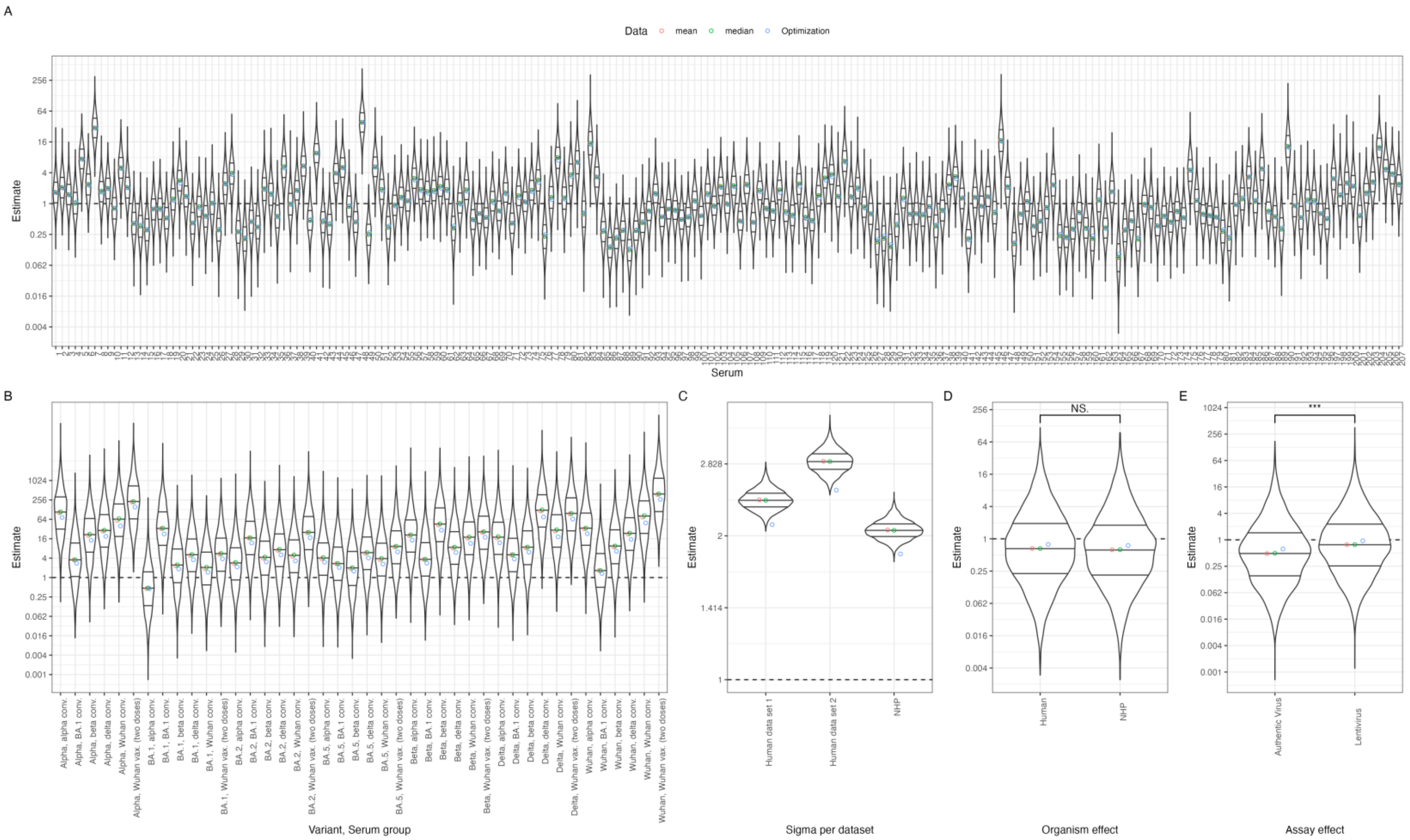

Figure S9. Bayesian modelling estimates, related to Figure 4. Estimated **A.** serum reactivity effect, **B.** underlying variant titer/10 per serum group, **C.** normally distributed error (sigma) per dataset (Human data set 1 [3], Human data set 2 [4] and NHP), **D.** organism and **E.** assay effects from 4000 independent draws. The mean and median of the draws are shown in red and green, respectively, and the mean from optimizing the model is shown in blue. **Table S4** links serum number in **A.** and serum name. Estimated **D.** organism and **E.** assay effects from independent were compared using a T-test, normality was checked with a Shapiro-Wilk test (Human p=0.224, NHP p=0.352, Authentic virus p=0.085, Lentivirus p=0.304). No significant difference was detected for organism effects (statistic = 1.52, df = 7998, p = 0.128). The difference between assay effects was highly significant (statistic = -13, df = 7988, p = 2.78e-38).

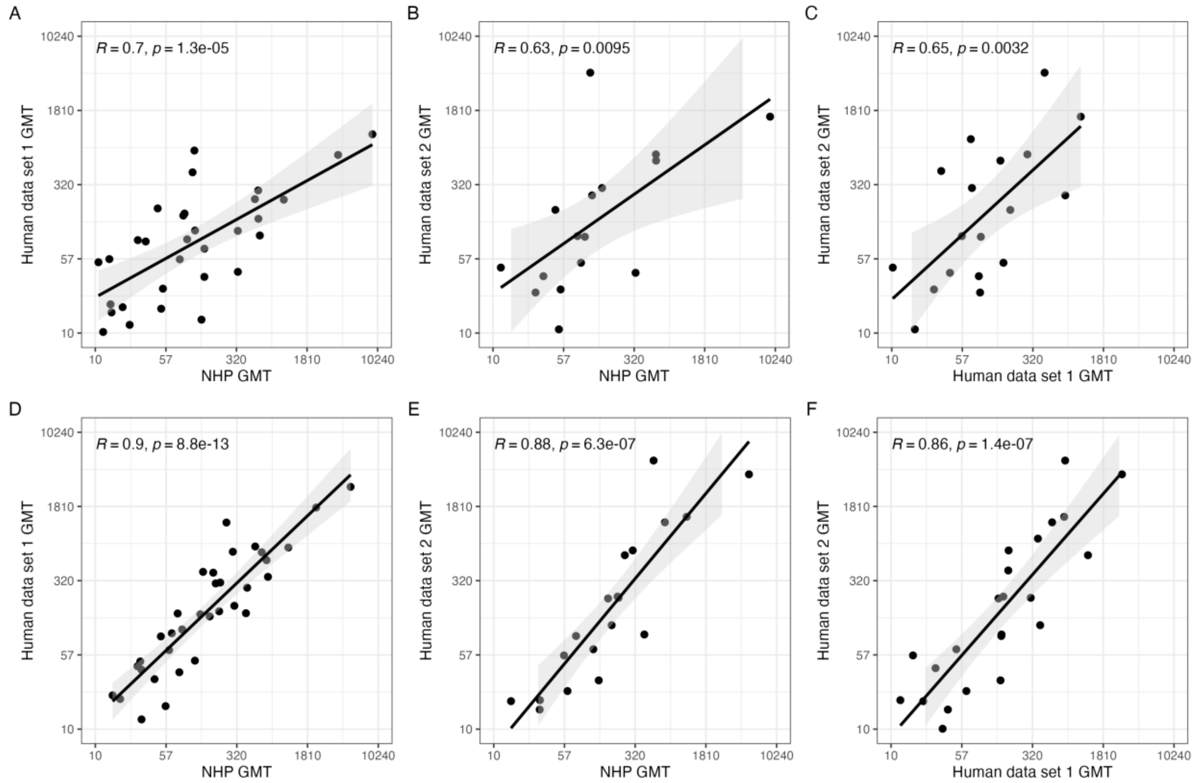

Figure S10. GMT correlation of data sets, related to Figure 4. A-C Correlation of GMTs calculated from imputed titers across datasets before adjusting for assay and organism effects and **D-E** after adjusting for assay and organism effects. The Pearson correlation coefficient is shown for NHP vs. Human data set 1 [3] (**A, D**), NHP vs. Human data set 2 [4] (**B, E**) and Human data set 1 vs. Human data set 2 (**C, F**).

Supplementary Tables

Table S1. SARS-CoV-2 variants analyzed in the pseudovirus neutralization assay, related to Figure 1

**Category**	**Virus variant (Pango lineage)**	**GISAID ID†**
Pre-Omicron variants	Wuhan (WA1/2020)	EPI_ISL_402124
	B.1.1.7	EPI_ISL_601443
	B.1.351	EPI_ISL_712096
	B.1.617.2	EPI_ISL_2020950
Early Omicron variants	BA.1	EPI_ISL_7358094.2
	BA.2	EPI_ISL_6795834.2
	BA.2.12.1	EPI_ISL_12003853.1
	CH.1.1	EPI_ISL_17202004
	DV.7.1	EPI_ISL_18052118
	BA.5	EPI_ISL_12268495.2
	BQ.1.1	EPI_ISL_14752457
XBC.1.6 and XBB.1-descended variants	XBB.1	EPI_ISL_15232105
	XBB.1.5	EPI_ISL_16418320
	XBC.1.6	EPI_ISL_17851490
	FL.1.5.1	EPI_ISL_18126515
	HV.1	EPI_ISL_18592608
	HK.3	EPI_ISL_18631954
	EG.5.1	EPI_ISL_17976635
Recent saltation Omicron variants	BA.2.86	EPI_ISL_18121060
	JN.1	EPI_ISL_18680594
	KP.2	EPI_ISL_19189039
	KP.3	EPI_ISL_19203968
	KZ.1.1.1	EPI_ISL_19028189

†The spike sequences for the pseudovirus generation were retrieved from the GISAID Initiative [5].

Table S2. Characteristics of infected NHP study cohorts, related to Figures 1 and 2

**Study cohort**	**Virus variant of challenge (Pango lineage)**	**SARS-CoV-2 isolate**	**GISAID ID**	**Number of animals (n)**	**Blood collection***
**Wuhan**	Wuhan	hCoV-19/USA-WA1/2020	EPI_ISL_404895.2	5	20#
**Alpha**	B.1.1.7	hCov-19/USA/CA_CDC_5574/2020	EPI_ISL_751801	6	21#
**Beta**	B.1.351	hCov-19/South Africa/KRISP-K005325/2020	EPI_ISL_678615	12	10 (n=6)<br>21 (n=6#)
**Gamma**	P.1	hCoV-19/Japan/IC-0564/2021	EPI_ISL_792683	6	10
**Delta**	B.1.617.2	hCoV-19/USA/MD-HP05647/2021	EPI_ISL_2331496	16	10
**BA.1**	B.1.1.529	hCoV-19/USA/MD-HP20874/2021	EPI_ISL_7160424	11	10 (n=6)<br>28 (n=5#)
**BA.2.12.1**	BA.2.12.1	hCoV-19/USA/NY-MSHSPSP-PV56475/2022	EPI_ISL_11685455	3	10
**BA.4**	BA.4	hCoV-19/USA/MDHP30386/2022	EPI_ISL_12416220	3	10
**BA.5**	BA.5	hCov-19/USA/COR-22-063113/2022	EPI_ISL_13512579	9	10

*Interval between virus challenge and blood collection is given in days.
#Additional samples from an earlier timepoint (day 10 post infection) have been analyzed (**figure S4**).

Table S3. Characteristics of vaccinated NHP study cohorts, related to Figures 1 and 2

Study cohort	Number of animals (n)	COVID-19 vaccine	Vaccine dose	Vaccine doses† (n)	Blood collection*
**Wuhan**	4	Ad.26.COV.S; Janssen COVID-19 viral-vector vaccine	$5x10^{10}$ Virus particles	1	26
**Beta**	4	Ad.26.COV.S.351; Janssen COVID-19 viral-vector vaccine	$5x10^{10}$ Virus particles	1	26
**Wuhan**	6	BNT162b2; Pfizer COVID-19 mRNA vaccine	30 ug mRNA	2	14
**XBB.1.5**	6	BNT162b2; Pfizer COVID-19 mRNA vaccine	30 ug mRNA	2	14

†Number of subsequent homologous vaccine doses.
*Interval between last vaccination and blood collection is given in days.

Table S5. Number of titrations per serum group, variant and data set used in the Bayesian comparison, related to Figure 4. Titrations from maps by Roessler *et al.* [3] (Human data set 1) and Wilks *et al.* [4] (Human data set 2) that were common in serum group and variant with the NHP data set were extracted. The D614G variant in the human data set was taken as Wuhan equivalent.

Serum group	Virus variant	Human data set 1	Human data set 2	NHP
BA.1 infected	Alpha	14	1	11
BA.1 infected	BA.1	14	4	11
BA.1 infected	BA.2	14	4	11
BA.1 infected	BA.5	14	4	11
BA.1 infected	Beta	14	4	11
BA.1 infected	Delta	14	1	11
BA.1 infected	Wuhan	14	4	11
Wuhan infected	Alpha	10	12	5
Wuhan infected	BA.1	10	13	5
Wuhan infected	BA.2	10	13	5
Wuhan infected	BA.5	10	0	5
Wuhan infected	Beta	10	13	5
Wuhan infected	Delta	10	12	5
Wuhan infected	Wuhan	10	13	5
Wuhan vaccinated (two doses)	Alpha	11	26	6
Wuhan vaccinated (two doses)	BA.1	11	13	6
Wuhan vaccinated (two doses)	BA.2	11	0	6
Wuhan vaccinated (two doses)	BA.5	11	4	6
Wuhan vaccinated (two doses)	Beta	11	28	6
Wuhan vaccinated (two doses)	Delta	11	26	6
Wuhan vaccinated (two doses)	Wuhan	11	30	6
Alpha infected	Alpha	9	13	6
Alpha infected	BA.1	9	13	6
Alpha infected	BA.2	9	13	6
Alpha infected	BA.5	9	0	6
Alpha infected	Beta	9	13	6
Alpha infected	Delta	9	13	6
Alpha infected	Wuhan	9	13	6

Beta infected	Alpha	6	15	12
Beta infected	BA.1	6	15	12
Beta infected	BA.2	6	0	12
Beta infected	BA.5	6	0	12
Beta infected	Beta	6	15	12
Beta infected	Delta	6	15	12
Beta infected	Wuhan	6	15	12
Delta infected	Alpha	5	15	16
Delta infected	BA.1	5	21	16
Delta infected	BA.2	5	11	16
Delta infected	BA.5	5	6	16
Delta infected	Beta	5	21	16
Delta infected	Delta	5	21	16
Delta infected	Wuhan	5	21	16

Table S6. Significant differences between NHP and human titers after adjusting for modelled effects, related to Figure 4. Log2 titers after adjusting for serum, assay and organism effects were tested for normality with a Shapiro-Wilk test. Many titers differed significantly from a normal distribution and a Wilcox rank sum test was used to compare human and NHP titers per serum group and variant. Significant ($p < 0.05$) results are shown. Calculations were performed using the rstatix package (v 0.7.2)[6].

Serum group	Variant	group1	group2	n1	n2	statistic	p-value	Significance level
Wuhan infected	Beta	Human	NHP	23	5	6	0.00059	***
Wuhan infected	Delta	Human	NHP	22	5	100	0.0028	**
Wuhan vaccinated (two doses)	BA.1	Human	NHP	24	6	30	0.0286	*
Wuhan vaccinated (two doses)	Delta	Human	NHP	37	6	170	0.038	*
Wuhan vaccinated (two doses)	Wuhan	Human	NHP	41	6	58	0.0374	*
alpha infected	BA.1	Human	NHP	22	6	28	0.0331	*
beta infected	BA.1	Human	NHP	21	12	56	0.00785	**
beta infected	Beta	Human	NHP	21	12	232	1.46e-05	****
beta infected	Delta	Human	NHP	21	12	72	0.0441	*
delta infected	Alpha	Human	NHP	20	16	34	1.65e-05	****
delta infected	BA.5	Human	NHP	11	16	131	0.0343	*
delta infected	Beta	Human	NHP	26	16	64	8.96e-05	****
delta infected	Delta	Human	NHP	26	16	365	1.41e-05	****
BA.1 infected	Alpha	Human	NHP	15	11	33	0.00915	**
BA.1 infected	Beta	Human	NHP	18	11	54	0.0442	*

References

[S1] Wilks, S.H. (2022). titertools: A statistical toolkit for the annalysis of censored titration data.

[S2] Wilks, S.H. (2022). Racmacs: R Antigenic Cartography Macros.

[S3] Rössler, A., Netzl, A., Knabl, L., Bante, D., Wilks, S.H., Borena, W., von Laer, D., Smith, D.J., and Kimpel, J. (2023). Characterizing SARS-CoV-2 neutralization profiles after bivalent boosting using antigenic cartography. Nat Commun 14, 5224, https://doi.org/10.1038/s41467-023-41049-4.

[S4] Wilks, S.H., Mühlemann, B., Shen, X., Türeli, S., LeGresley, E.B., Netzl, A., Caniza, M.A., Chacaltana-Huarcaya, J.N., Corman, V.M., Daniell, X. et al. (2023). Mapping SARS-CoV-2 antigenic relationships and serological responses. Science 382, eadj0070, https://doi.org/10.1126/science.adj0070.

[S5] Khare, S., Gurry, C., Freitas, L., Schultz, M.B., Bach, G., Diallo, A., Akite, N., Ho, J., Lee, R.T., Yeo, W. et al. (2021). GISAID's Role in Pandemic Response. China CDC Wkly 3, 1049-1051, https://doi.org/10.46234/ccdcw2021.255.

[S6] Kassambara, A. (2023). rstatix: Pipe-Friendly Framework for Basic Statistical Tests.
